# *Mycobacterium tuberculosis*-Specific T Cell Functional, Memory, and Activation Profiles in QuantiFERON-Reverters Are Consistent With Controlled Infection

**DOI:** 10.3389/fimmu.2021.712480

**Published:** 2021-08-30

**Authors:** Cheleka A. M. Mpande, Pia Steigler, Tessa Lloyd, Virginie Rozot, Boitumelo Mosito, Constance Schreuder, Timothy D. Reid, Nicole Bilek, Morten Ruhwald, Jason R. Andrews, Mark Hatherill, Francesca Little, Thomas J. Scriba, Elisa Nemes

**Affiliations:** ^1^South African Tuberculosis Vaccine Initiative, Institute of Infectious Disease and Molecular Medicine, Division of Immunology, Department of Pathology, University of Cape Town, Cape Town, South Africa; ^2^Wellcome Centre for Infectious Diseases Research (CIDRI) in Africa, Institute of Infectious Disease and Molecular Medicine and Division of Immunology, Department of Medicine, University of Cape Town, Cape Town, South Africa; ^3^Department of Statistical Sciences, University of Cape Town, Cape Town, South Africa; ^4^Statens Serum Institut, Copenhagen, Denmark; ^5^Foundation of Innovative New Diagnostics, Geneva, Switzerland; ^6^Department of Medicine, Stanford University, Stanford, CA, United States

**Keywords:** QuantiFERON reversion, memory T cell, donor unrestricted T cells, innate immune response, *Mycobacterium tuberculosis* infection

## Abstract

Reversion of immune sensitization tests for *Mycobacterium tuberculosis* (M.tb) infection, such as interferon-gamma release assays or tuberculin skin test, has been reported in multiple studies. We hypothesized that QuantiFERON-TB Gold (QFT) reversion is associated with a decline of M.tb-specific functional T cell responses, and a distinct pattern of T cell and innate responses compared to persistent QFT+ and QFT- individuals. We compared groups of healthy adolescents (n=~30 each), defined by four, 6-monthly QFT tests: reverters (QFT+/+/-/-), non-converters (QFT-/-/-/-) and persistent positives (QFT+/+/+/+). We stimulated peripheral blood mononuclear cells with M.tb antigens (M.tb lysate; CFP-10/ESAT-6 and EspC/EspF/Rv2348 peptide pools) and measured M.tb-specific adaptive T cell memory, activation, and functional profiles; as well as functional innate (monocytes, natural killer cells), donor-unrestricted T cells (DURT: γδ T cells, mucosal-associated invariant T and natural killer T-like cells) and B cells by flow cytometry. Projection to latent space discriminant analysis was applied to determine features that best distinguished between QFT reverters, non-converters and persistent positives. No longitudinal changes in immune responses to M.tb were observed upon QFT reversion. M.tb-specific Th1 responses detected in reverters were of intermediate magnitude, higher than responses in QFT non-converters and lower than responses in persistent positives. About one third of reverters had a robust response to CFP-10/ESAT-6. Among those with measurable responses, lower proportions of T_SCM_ (CD45RA+CCR7+CD27+) and early differentiated (CD45RA-) IFN-γ-TNF+IL-2- M.tb lysate-specific CD4+ cells were observed in reverters compared with non-converters. Conversely, higher proportions of early differentiated and lower proportions of effector (CD45RA-CCR7-) CFP10/ESAT6-specific Th1 cells were observed in reverters compared to persistent-positives. No differences in M.tb-specific innate, DURT or B cell functional responses were observed between the groups. Statistical modelling misclassified the majority of reverters as non-converters more frequently than they were correctly classified as reverters or misclassified as persistent positives. These findings suggest that QFT reversion occurs in a heterogeneous group of individuals with low M.tb-specific T cell responses. In some individuals QFT reversion may result from assay variability, while in others the magnitude and differentiation status of M.tb-specific Th1 cells are consistent with well-controlled M.tb infection.

## Introduction

Immunodiagnostics for *Mycobacterium tuberculosis* (M.tb) infection, such as tuberculin skin tests (TSTs) and IFN-γ release assays (IGRAs), were designed and are clinically interpreted based on the premise that detectable M.tb-specific immune responses are indicative of viable M.tb infection, i.e. bacterial persistence. Thus, asymptomatic M.tb infection in the absence of disease progression is commonly thought to be a chronic condition that may affect up to a quarter of the global population (1). However, longitudinal studies have demonstrated the dynamic nature of M.tb-specific immune responses, whereby TSTs and IGRAs revert from a positive to a negative test in some individuals.

Studies conducted during the pre-antibiotic era demonstrated that TST reversion was associated with a lower risk of disease progression. Detection of calcified lung lesions in some of these TST negative individuals, who had no recorded history of tuberculosis, was suggestive of contained or cleared disease (2). Guinea pigs that were infected with M.tb (defined by recent TST conversion upon experimental exposure) and then reverted to a negative TST had sterile lung lesions indicative of cured infection (3–5). These findings provide evidence that some individuals can spontaneously cure M.tb infection and gave rise to the hypothesis that reversion of immunodiagnostic test results may be associated with M.tb clearance. Reversion has been observed naturally (2, 6–14) and in association with antibiotic treatment (8, 13, 15, 16). Furthermore, a recent Bacille Calmette-Guerin (BCG) revaccination trial in M.tb-uninfected adolescents demonstrated vaccine efficacy against sustained M.tb infection, which was associated with transient IGRA conversion followed by reversion (IGRA- → IGRA+ → IGRA-) within a 6 month period (17). The phenomenon of IGRA reversions has posed challenges to clinicians making decisions about the need for preventive therapy, for which there is currently no guidance. Furthermore, as IGRA conversion is increasingly used as an outcome in vaccine trials, correctly interpreting reversions will be important to understanding vaccine efficacy.

IFN-γ, a T helper 1 (Th1) CD4 T cell associated cytokine, plays a critical role in the immune response to M.tb and is expressed by most M.tb-specific T cells (18, 19). Although many cell types have the capacity to produce IFN-γ, such as natural killer (NK) cells and donor-unrestricted T cells (DURT cells), including γδ T cells, mucosal-associated invariant T (MAIT) and natural killer T-like cells, IFN-γ expression by CD4 T cells is necessary for M.tb control (19, 20). Stimulation with M.tb CFP-10/ESAT-6 peptides in the IGRA primarily induces IFN-γ expression by MHC-class II restricted CD4 T cells (21), while DURT and NK cells contribute more significantly (approximately 50%) to IFN-γ production in response to whole mycobacteria, which also include non-protein antigens (22).

Functional and phenotypic characteristics of T cells, specifically CD4 T cells, are known to track antigen burden (23). We have recently demonstrated that relative proportions of M.tb lysate (cross-reactive with BCG)-specific IL-2+ and TNF+ stem cell memory (T_SCM_) and central memory (T_CM_) cells are higher in IGRA- compared to IGRA+ individuals, suggesting that low or no *in vivo* M.tb antigen exposure is associated with higher proportions of early differentiated mycobacteria-specific T cell subsets (24). These findings are supported by a study on resisters, tuberculosis household contacts (HHC) who remain TST and IGRA negative, showing that these individuals have detectable M.tb-specific antibodies and low levels of IFN-γ-independent (CD154+TNF+IL-2+) CD4 T cell responses (25). Expression of CD154, TNF and IL-2 is a hallmark of quiescent T_SCM_ and T_CM_ cells, which maintain long-lasting immunity in the absence of antigen exposure (23, 26). Further, we and others have demonstrated that high T cell activation, measured by CD38, HLA-DR or Ki-67 expression, is associated with recent M.tb infection, high risk to tuberculosis progression, or on-going tuberculosis disease (27–32). These data suggest that levels of M.tb-specific T cell activation are likely associated with M.tb antigen load, which in animal models peaks during primary infection, decreases to a plateau during established infection, and increases again during disease (33).

Acknowledging that measuring *in vivo* M.tb load in humans is not possible, we hypothesized that if reversion is associated with controlled infection this would be reflected in immune features associated with antigen load. Specifically, we would expect an increase in the relative proportions of M.tb-specific cells with T_SCM_ and T_CM_ phenotypes, IFN-γ-independent (CD154+, IL-2+ and/or TNF+) functional responses and lower T cell activation in reverters compared to persistent QFT+ individuals. Since magnitude and functional profiles of antigen-specific T cells can be modulated by their interaction with innate cells, we further hypothesized that reverters would have reduced expression of pro-inflammatory cytokines (IFN-γ, IL-6 and/or TNF), increased expression of regulatory cytokines (IL-10) and/or reduced Th1 polarization (IL-12) by innate immune cells compared to persistently QFT+ individuals.

To test our hypotheses, we measured memory and functional profiles of M.tb-specific T cells, and functional features of myeloid, NK, DURT and B cells in persistent IGRA+ individuals, reverters and non-converters. We further integrated features from both adaptive and innate immune responses and applied statistical modelling to define the relationship between IGRA reverters, persistent IGRA+ individuals and non-converters.

## Methods

### Study Design

South African adolescent participants were selected from a large epidemiological study conducted in the Worcester region of Western Cape, South Africa, between July 2005 and February 2009 [University of Cape Town Human Research Ethics Committee protocol references: 045/2005, 102/2017; (34)]. All adolescents were assumed to be BCG vaccinated at birth, according to the South African expanded program of immunization. M.tb infection status was determined using QuantiFERON-TB Gold In-Tube (QFT, Qiagen) and peripheral blood mononuclear cells (PBMC) were collected at enrolment and at 6-monthly intervals during 2 years of follow-up in a subset of the cohort. TST was also performed at yearly intervals. Adolescent participants provided written, informed assent and their parents or legal guardians provided written, informed consent. All participants were between 12-18 years old and enrolled at public high schools. Participants who were pregnant, lactating or who had chronic medical conditions at enrolment were excluded. We retrieved stored PBMC from healthy adolescents based on serial QFT test results, as defined below, and sample availability.

### Definition of Study Groups

*QFT reverters* were defined as adolescents with two positive QFT tests followed by two negative QFT tests 6 months apart (**Supplementary Figure 1**). To reduce the likelihood of technical fluctuations around the assay cut-off, we selected reverters with at least one QFT positive and one negative test result outside the QFT uncertainty zone: 0.2-0.7 IU/mL [**Supplementary Figure 1**; (35)], wherever possible.

*Persistent QFT+* individuals had 4 consecutive QFT positive tests, at least two of which with IFN-γ >0.7 IU/mL, 6 months apart over 18 months. This group has been described in detail in [(24, 36); **Supplementary Figure 1**].

*QFT non-converter* adolescents had 4 consecutive QFT negative tests (< 0.2 IU/mL) 6 months apart (**Supplementary Figure 1**). QFT non-converters were not selected based on TB exposure history, in this high transmission setting, and likely include a heterogeneous population of naïve participants, as well as previous TB exposed but uninfected individuals. Only 4 non-converters reported past TB exposure (all more than 1 year prior to study enrolment, **Supplementary Table 1**). We chose an upper limit of QFT response of 0.2 IU/mL, below the QFT uncertainty zone, to increase the likelihood that these individuals were truly M.tb “unsensitized” at the time of sampling, and could represent a meaningful negative control group.

Control groups (non-converters and persistent QFT+) were randomly selected from a pool of participants with available samples and matched to QFT reverters based on age at enrolment (± 1 years), gender, ethnicity and school [which relates to socio-economic and M.tb exposure status (37), **Supplementary Table 1**].

### Measurement of M.tb-Reactive Adaptive and Innate Cells

M.tb-reactive adaptive (T cells) and innate cells [monocytes, NK, DURT and B cells (in their antigen-presenting cell capacity)] were detected using two different PBMC intracellular cytokine staining (ICS) and flow cytometry protocols.

The adaptive T cell protocol has been previously described in detail (24). Briefly, cryopreserved PBMC were stimulated with no antigen, peptide pools (15 mer peptides overlapping by ten amino-acids, 1μg/mL) spanning the full length of CFP-10/ESAT-6 (antigens in the QFT assay, GenScript Biotechnology) or Rv3615c (EspC) (33), Rv2348 and Rv3865 (EspF) (collectively referred to as EspC/EspF/Rv2348 peptide pool, GenScript Biotechnology), M.tb lysate (H37Rv, 10μg/mL, BEI resources) or Staphylococcus enterotoxin B [SEB (positive control), 1μg/mL, Sigma Aldrich] for 18 hours, with brefeldin A (5μg/mL, Sigma Aldrich) and monensin (2.5μg/mL, Sigma Aldrich) added after the first 3 hours of stimulation.

For the innate PBMC-ICS protocol, cryopreserved PBMC were stimulated with no antigen, M.tb lysate (H37Rv, 10μg/mL, BEI resources) or heat-killed Escherichia coli (*E.coli*, 10^7^ bacteria per 10^6^ PBMC, in-house preparation) for 6 hours, with brefeldin A (5μg/mL, Sigma Aldrich) and monensin (2.5μg/mL, Sigma Aldrich) added after the first 2 hours of stimulation (see **Supplementary Methods** for details).

Cells were then stained with fluorescent-labelled antibodies (**Supplementary Tables 2, 3**) and detected using flow cytometry to identify phenotypic marker and cytokine expression.

### Data Analysis

#### Adaptive T Cell Analysis Pipeline

M.tb-specific lymphocytes, CD4 and CD8 T cells stimulated using the adaptive T cell protocol were identified using the gating strategy illustrated in **Supplementary Figure 2**. Most analyses from the adaptive T cell protocol focused on CD4 T cells because CD8 T cell responses were predominantly not different to background response in most individuals (data not shown). We then followed the analysis pipeline previously described (24): we utilized COMPASS (38), Pestle and SPICE (39) to analyze expression of all antigen-specific cytokine combinations. We then identified individuals, referred to as responders, with total IFN-γ+ lymphocyte or Th1 cytokine+ CD4 T cell responses significantly [false discovery rate (FDR) ≤ 0.01] higher than background (unstimulated) using MIMOSA (40) and a fold-change over background ≥ 3. Since we had longitudinal samples, we applied the responder definition to each study visit for each participant. To prevent the introduction of bias, we first determined if frequencies of IFN-γ+ lymphocytes or Th1 cytokine+ CD4 T cells were significantly different between visits stratified by QFT status (detailed in Supplementary methods; **Supplementary Figure 3**). We then exported FCS files of IFN-γ+ lymphocytes or Th1 cytokine+ CD4 T cells from all visits that passed the responder criteria and concatenated FCS files based on QFT status to get a representative FCS file for each participant-QFT status combination (**Supplementary Figure 4**). Concatenated FCS files containing IFN-γ+ lymphocytes were used for tSNE analysis to identify cell clusters contributing to the total IFN-γ+ lymphocyte response (detailed in Supplementary Methods). Th1 cytokine+ CD4 T cells were used for CITRUS analysis to identify unique T cell features that could distinguish between two groups, *i.e.* persistent QFT+ *versus* pre-reverter, pre- *versus* post-reverter and post-reverter *versus* non-converter [**Supplementary Figure 5**; (41)]. We also used Th1 cytokine CD4 T cell responses to calculate functional differentiation score [**Supplementary Equation 1**; (42)].

#### Innate Cell Analysis Pipeline

M.tb-specific innate (monocytes and NK cells), DURT, B cells and T cells stimulated using the innate PBMC-ICS protocol were identified using the gating strategy illustrated in **Supplementary Figure 6**. Since spontaneous cytokine expression was detected in several innate cell subsets, as expected, analyses included responses measured in unstimulated samples, stimulated samples, as well as stimulated minus unstimulated samples. Since our flow cytometry panel included several functions that are known not to be expressed by all the cell subsets measured (i.e. are biologically irrelevant) we excluded variables that were not measurable in two-thirds of the samples [details about data filtering are provided in (43)]. Additional information about tSNE analyses of this dataset is provided in the **Supplementary Methods**.

#### Statistical Analysis

The adaptive T cell experimental protocol was performed on all study participant visits, where possible, while the innate protocol was performed on PBMC from all reverters visits, were possible, but on only two (consecutive) visits from persistent QFT+ individuals and non-converters. To prevent the introduction of bias due to repeated sampling from the same donor, we calculated a representative functional response based on QFT status for each individual. Firstly, to determine if functional responses (single cytokine, co-expression patterns, etc.) were significantly different between visits stratified by QFT status, we performed a Kruskal-Wallis test to compare responses detected at all 4 QFT negative and positive visits in non-converters and persistent QFT+, respectively (**Supplementary Figures 3A, B**). Wilcoxon signed-rank tests were used to compare functional responses detected at paired QFT negative and QFT positive visits in reverters (**Supplementary Figure 3C**). If none of the functional responses were significantly different (p > 0.05) between visits with the same QFT status, we calculated the median response for each participant based on QFT status. This resulted in a single value for each QFT non-converter and persistent QFT+ individual, and two values for QFT reverters according to QFT status (QFT+ = pre-reverter and QFT- = post-reverter), which were then used for inter- and intra-group comparisons: persistent QFT+ *vs* pre-reverter (Mann-Whitney test); pre- *vs* post-reverter (Wilcoxon signed-rank test) and post-reverter *vs* non-converter (Mann-Whitney test).

Bonferroni and Benjamini-Hochberg (FDR <0.05) methods were used to correct for multiple comparisons for up to 4 comparisons or more than 4 comparisons, respectively.

#### Projection to Latent Space Discriminant Analysis Modelling Pipeline

Several data pre-processing steps were applied prior to building the projection to latent space discriminant analysis (PLS-DA) (30) model, detailed in the **Supplementary Methods**.

##### Feature Selection

We tuned and built a least absolute shrinkage and selection operator (LASSO) model (44) to the integrated dataset in order to identify features that could stratify the two control groups (persistent QFT+ and non-converters) in the model. The model was therefore blinded to the differences between the reverters and the control cohorts. 10-fold cross-validation (CV) was repeated 500 times and the optimal shrinkage parameter, λ, was defined as the average λ across the 500 repeats. The final LASSO model was built using the optimal value of λ and the most stratifying features were identified as the non-zero coefficients in the final model.

##### PLS-DA

PLS-DA is a classification model that aims to classify samples into known groups and identify variables that drive the discrimination between the groups. The PLS-DA model (45) was built within the *ropls* R package (R package version 1.22.0. doi:10.18129/B9.bioc.ropls) to the vast standardized and MFA-imputed dataset containing the features selected by the LASSO model. The group status, namely persistent QFT positive, pre-reversion, post-reversion and non-converter, was defined as the response.

##### Model Validation

The performance of the PLS-DA model was assessed *via* a cross-validation procedure. For 1000 bootstrapped samples, 70% of the observations were set aside to make up the training set, and the remaining 30% the testing set. For each iteration, the data was split such that there were equal proportions of the four groups in the testing set (10 observations per group). The PLS-DA model was built to the training set of observations and the test set was used to assess the ability of the model to predict a new outcome based on its training. The performance was reported as the average misclassification error across the 1000 bootstrapped iterations.

## Results

### TST Dynamics

We defined M.tb infection using QFT only, but also had access to TST results, which were performed annually. We therefore determined the concordance between QFT and TST for each study group. We observed very good concordance of 95% and 88% between QFT and TST in persistent QFT+ individuals and non-converters, respectively (**Supplementary Figure 7**; **Supplementary Table 4**). However, the majority of QFT reverters were persistently TST+ throughout follow up, which resulted in a poor concordance between QFT and TST tests, with a 45% agreement between the two immuno-diagnostics (**Supplementary Figure 7**; **Supplementary Table 4**).

### QFT Reversion Is Associated With Maintenance of Functional M.tb-Specific CD4+ T Cells

We utilized tSNE analysis to determine the cellular composition of CFP-10/ESAT-6- and M.tb lysate-specific IFN-γ+ lymphocytes in responders only. Too few reverters and non-converters had a robust IFN-γ+ lymphocyte response to CFP-10/ESAT-6 to be included in this analysis (**Supplementary Table 5**). tSNE analysis (and confirmatory flow cytometry gating) confirmed CD4 T cells as the major source of IFN-γ in persistent QFT+ individuals (regardless of stimuli) and (pre- and post-) reversion, while CD4 T cells accounted for <50% of M.tb lysate-specific IFN-γ+ lymphocytes in non-converters (**Supplementary Figure 8**). Based on these results, we decided to focus our T cell analysis on CD4 T cells.

**Figure 1:**
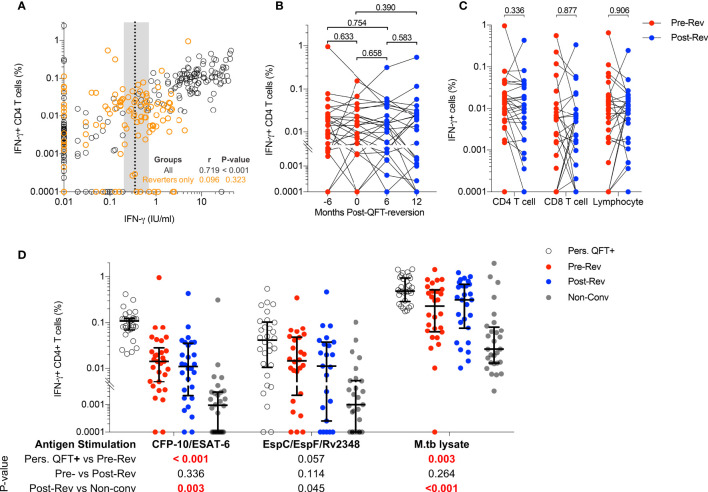
Immunodominant M.tb-specific IFN-γ+ CD4 T cells are maintained during QFT reversion. **(A)** Immune responses detected by flow cytometry (% of CFP-10/ESAT-6-specific IFN-γ+ CD4 T cells) and QFT-ELISA (IFN-γ+ IU/mL). All participants and all visits are shown (n = 321). Dotted line and shaded area represent the QFT cut-off (0.35 IU/mL) and the uncertainty zone (0.2-0.7 IU/mL), respectively. The Spearman’s correlation coefficient (r) was calculated on the entire cohort (black) and reverters only (orange). **(B)** Frequencies of background subtracted CFP-10/ESAT-6-specific IFN-γ+ CD4 T cells detected in QFT reverters at Month -6: n = 26 (QFT+, red symbol), Month 0: n = 25 (QFT+, red symbol), Month 6: n = 28 (QFT-, blue symbol) and Month 12: n = 26 (QFT-, blue symbol) post QFT reversions. **(C)** Median frequencies of CFP-10/ESAT-6-specific IFN-γ+ among CD4 and CD8 T cells and total lymphocytes, measured pre- (n = 30, red symbol) and post- (n = 28, blue symbol) QFT reversion. **(D)** CFP-10/ESAT-6- (P10-ES6), EspC/EspF/Rv2348- (Esp) and M.tb lysate- (M.tbL) specific IFN-γ+ CD4 T cells detected in persistent QFT+ individuals (P10-ES6: n = 29; Esp: n = 30; M.tbL: n = 30, white), pre-reversion (P10-ES6: n = 30; Esp: n = 26; M.tbL: n = 28, red), post-reversion (P10-ES6: n = 28; Esp: n = 23; M.tbL: n = 27, blue) and non-converters (P10-ES6: n = 28; Esp: n = 27; M.tbL: n = 28, grey). P-values were calculated using the Wilcoxon signed rank test for comparison of responses from reverters only, and Mann-Whitney test for persistent QFT+ *vs* pre-reversion and post-reversion *vs* non-converters. P-values < 0.05 for **(A)**, < 0.01 for **(B)** and < 0.0125 (for **C, D**) were considered significant after correction for multiple comparisons.

Our experimental protocol detected CFP-10/ESAT-6-specific IFN-γ+ CD4 T cells that highly correlated with QFT responses (**Figure 1A**), confirming that PBMC-ICS and flow cytometry were suitable techniques to characterize M.tb-specific immune responses detected by QFT. However, CFP-10/ESAT-6-specific IFN-γ+ CD4 T cells did not decrease upon QFT reversion, and frequencies of these cells were maintained at similar levels throughout the study follow-up and did not correlate with quantitative QFT values (**Figures 1A** **, B**). Some QFT reverters (n=8) had either both pre-reversion or post-reversion QFT values within the uncertainty zone (**Supplementary Figure 1**). Regardless, the frequencies of their CFP-10/ESAT-6-specific IFN-γ+ CD4 T cells fell in the same dynamic range and showed no change over time as participants with at least one QFT positive value above 0.7 IU/mL and one QFT negative value below 0.2 IU/mL (data not shown). Since we did not observe a difference in frequencies of IFN-γ+ CD4 T cells across different visits, we calculated median responses for each participant stratified by QFT status. No significant differences in CFP-10/ESAT-6-specific IFN-γ+ CD4 T cells, nor IFN-γ+ CD8 T cells or IFN-γ+ lymphocytes, were observed pre- and post-reversion (**Figure 1C**).

We then determined whether recognition of other M.tb-specific immunodominant antigens or expression of IFN-γ-independent cytokines were also maintained in reverters. EspC/EspF/Rv2348- and M.tb lysate-specific IFN-γ+ CD4 T cells did not significantly decrease upon QFT reversion (**Figure 1D**). Frequencies of CFP-10/ESAT-6- and M.tb lysate-specific IFN-γ+ CD4 T cells detected pre-reversion were significantly lower than those in persistent QFT+ individuals, while those detected post-reversion were significantly higher than non-converters, respectively (**Figure 1D**). On the other hand, the magnitude of EspC/EspF/Rv2348-specific IFN-γ+ CD4 T cells observed pre- and post-QFT reversion was similar to persistent QFT+ individuals and non-converters, respectively (**Figure 1D**), probably due to high variability. Lastly, as observed for IFN-γ+ CD4 T cells, frequencies of TNF+ and IL-2+ CFP10-/ESAT-6-specific CD4 T cells did not decrease upon QFT reversion, and responses detected in reverters were of intermediate magnitude between non-converters and persistent QFT+ (**Supplementary Figure 9A**). The magnitude of CD154+ and CD107+ CFP10-/ESAT-6-specific CD4 T cells was not different between groups. (**Supplementary Figure 9A**).

**Figure 2:**
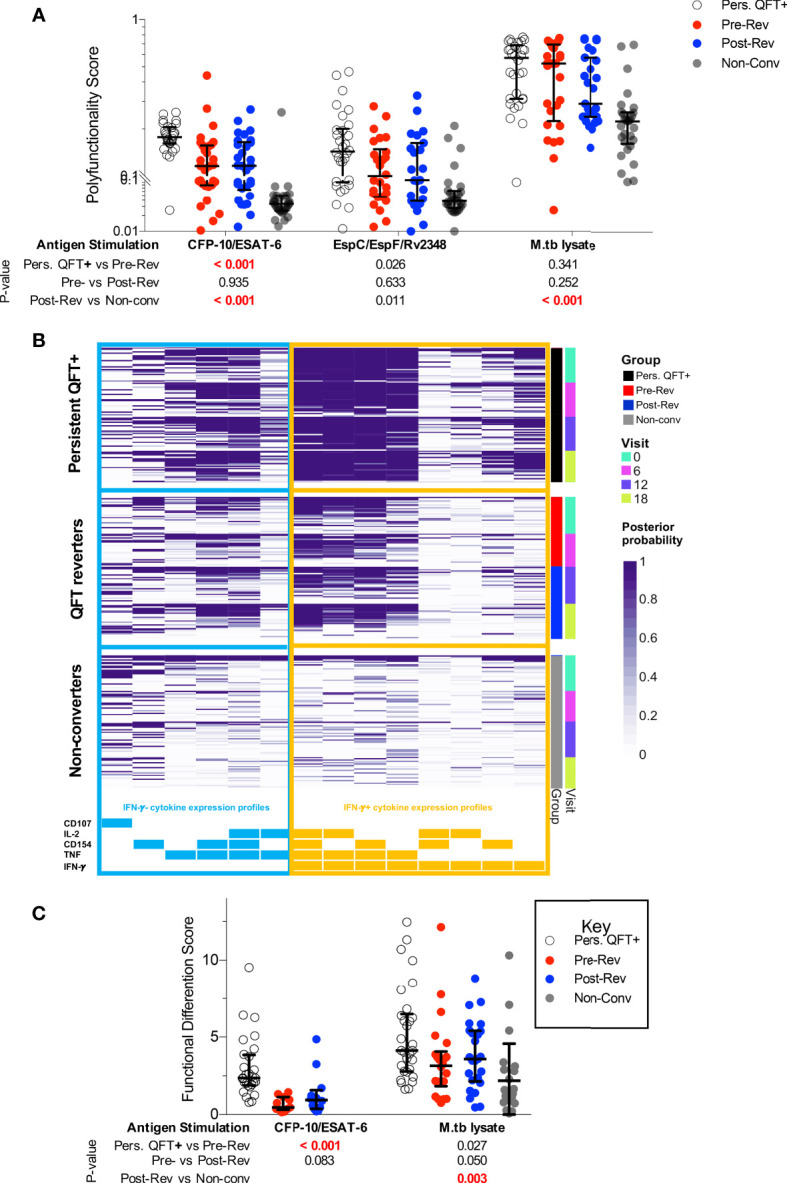
Functional profiles of M.tb-specific CD4 T cells are maintained during QFT reversion. **(A)** CFP-10/ESAT-6-, EspC/EspF/Rv2348- and M.tb lysate-specific polyfunctionality scores calculated by COMPASS. **(B)** Posterior probabilities (purple shading) of detecting CFP-10/ESAT-6-specific CD4 T cells expressing different combinations of functional markers, as indicated at the bottom. Subsets including IFN-γ- cytokine expression profiles are grouped on the left (blue) and those including IFN-γ+ subsets are shown on the right (orange). All participants from all study groups and visits are shown. **(C)** Functional differentiation score (**Supplementary Equation 1**) of M.tb-specific CD4 T cells in persistent QFT+ (P10-ES6: n = 28; M.tbL: n = 30), pre-reversion (P10-ES6: n = 11; M.tbL: n = 22), post-reversion (P10-ES6: n = 12; M.tbL: n = 25) and non-converters (M.tbL: n = 24). Number of participants for **(A, B)**, and calculation of P-values are as in **Figure 1**. P-values highlighted in bold red were considered significant.

Next, we investigated changes in M.tb-specific CD4 T cell cytokine co-expression profiles, and specifically whether QFT reversion was associated with preferential maintenance of IFN-γ-independent responses (observed in resisters) compared to persistent QFT+ individuals. The probability of detecting polyfunctional responses [polyfunctionality score calculated by COMPASS (38)] and frequencies of cytokine co-expressing CD4 T cell subsets were not significantly different pre- and post-reversion, with no enrichment of IFN-γ-CD154+TNF+IL-2+ CD4 T cells (**Figures 2A, B** and **Supplementary Figure 9B**). Group comparisons confirmed previous observations, where CFP-10/ESAT-6 polyfunctionality scores and the frequencies of IFN-γ+ co-expressing M.tb-specific cells in reverters were lower than persistent QFT+ individuals but higher than non-converters (**Figures 2A, B** and **Supplementary Figures 9B**). Exceptions to this were comparable M.tb lysate-specific polyfunctionality scores and frequencies of CFP-10/ESAT-6 IFN-γ- CD4 T cell subsets (including CD154+TNF+IL-2+) between persistent QFT+ individuals and pre-reverters (**Figures 2A, B** and **Supplementary Figure 9B**).

Further characterization of M.tb-specific CD4 T cells focused only on participants with robust Th1 responses (**Supplementary Table 6**), and only groups with at least one third of participants with a Th1 response were included. Definition of “responders” ignored expression of CD154 and CD107 because of their high background in unstimulated samples. Based on these arbitrary criteria, CFP-10/ESAT-6-specific responses in non-converters and EspC/EspF/Rv2348-specific responses in all groups were not further studied. Importantly, only 30-40% reverters had robust responses to CFP-10/ESAT-6, which were detectable in 97% of persistent QFT+ individuals.

Next, we compared proportions of Th1 cells expressing different combinations of IFN-γ, TNF and IL-2 in responders only. IFN-γ- CD4 T cells accounted for less than a quarter of M.tb lysate-specific CD4 T cells in persistent QFT+ individuals and reverters, compared to almost 40% of M.tb lysate-specific CD4 T cells in non-converters and only minor differences were observed across the study groups (**Supplementary Figure 10A**). On the other hand, more than half of CFP-10/ESAT-6-specific responses in reverters were IFN-γ-, compared to less than 30% in persistent QFT+ individuals. Additionally, pre-reverters exhibited higher proportions of CFP-10/ESAT-6-specific IFN-γ-TNF+IL-2±, but lower proportions of IFN-γ+TNF+IL-2± compared with persistent QFT+ (**Supplementary Figure 10A**). Overall, reverters had a lower functional differentiation score compared to persistent QFT+ individuals in response to CFP-10/ESAT-6 and a higher functional differentiation score compared to non-converters in response to M.tb lysate (**Figure 2C**).

Taken together, these results suggest that although functional responses to diverse M.tb antigens are maintained even upon QFT reversion, reverters have intermediate magnitudes of M.tb-specific CD4 T cells responses and these cells are in intermediate states of functional differentiation compared to persistent QFT+ and non-converters.

### QFT Reversion Is Not Associated With a Shift Towards a Less Differentiated T Cell Memory Phenotype

Less differentiated T cell memory and IFN-γ- functional phenotypes have been associated with lower antigen loads (23, 42). To determine if this is also true for M.tb-specific CD4 T cells during QFT reversion, we measured the proportions of M.tb-specific Th1 cells expressing different memory phenotypes in reverters and compared them to control groups. We defined memory subsets based on co-expression of CD45RA (RA), CCR7 (R7), CD27 (27) and KLRG-1 (G1). The predominant memory subsets in all groups, regardless of stimulation, were transitional (T_TM_) and effector (T_E_) memory T cells (**Supplementary Figure 10B**). We observed higher proportions of CFP-10/ESAT-6-specific T_CM_ cells in pre-reverters than in persistent QFT+ individuals, and lower proportions of M.tb lysate-specific T_SCM_ cells post-reversion compared with non-converters (**Supplementary Figure 10B**).

To obtain more granular results, we next evaluated whether a combination of memory and functional markers could be used to identify subsets that were significantly different between groups using an unbiased approach, CITRUS [as described in (24)]. The composition of each differentially expressed T cell cluster (**Supplementary Table 7**) identified using CITRUS, was confirmed by manual gating on all responders (**Supplementary Figure 5B**).

**Figure 3:**
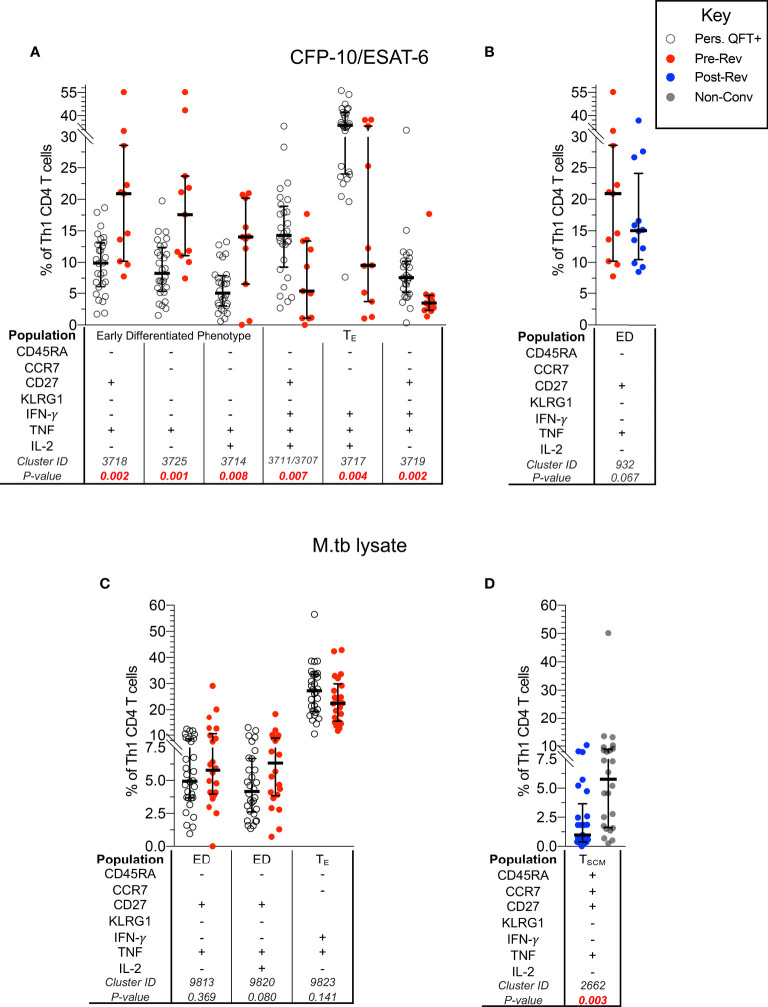
CFP-10/ESAT-6-specific memory/functional co-expression patterns are different between persistent QFT+ and reverters. Scatter plots (with medians and inter-quartile ranges) of manually gated memory and functional markers combinations representing CITRUS clusters that were different between: CFP-10/ESAT-6-specific CD4 T cells in **(A)** persistent QFT+ *versus* pre-reverters and **(B)** pre- *versus* post-reverters, and M.tb lysate-specific CD4 T cells in **(C)** persistent QFT+ *versus* pre-reverters and **(D)** post-reverters *versus* non-converters. Number of participants, confirmatory statistical analysis and p-value calculations is as in **Figure 2C**.

In line with previous analyses, pre-reverters showed higher proportions of CFP-10/ESAT-6-specific early differentiated IFN-γ- Th1 cells, and lower proportions of more differentiated effector IFN-γ+ Th1 cells compared to persistent QFT+ individuals (**Figure 3A**).

Despite detecting differences in cellular composition using CITRUS, proportions of manually gated CFP-10/ESAT-6-specific early differentiated CD27+TNF+ Th1 cells (cluster 932) were not significantly different pre- *versus* post-reversion (p = 0.067; **Figure 3B**). Similarly, the 3 M.tb lysate-specific clusters identified by CITRUS as differentially expressed in persistent QFT+ compared to pre-reverters were not different when gated manually (p > 0.05; **Figure 3C**). On the other hand, we confirmed lower proportions of M.tb lysate-specific TNF+ T_SCM_ (cluster 2662) cells in post-reverters compared to non-converters (**Figure 3D**), while no other differences were detected between these 2 groups.

Based on these results, we can conclude that M.tb-specific CD4+ T cells from QFT reverters are less differentiated compared to persistent QFT individuals, and are more similar to non-converters.

### QFT Reversion Is Not Associated With a Decrease in T Cell Activation

**Figure 4:**
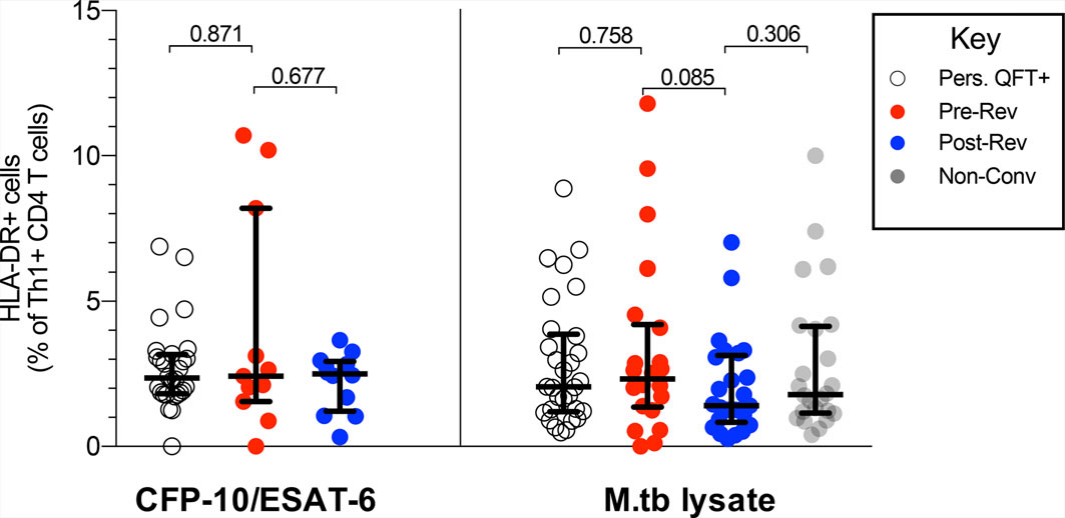
QFT reversion is not associated with a decrease in T cell activation. Graphs depict proportions of HLA-DR+ CFP-10/ESAT-6- and M.tb lysate- specific Th1 cytokine+ CD4 T cells in persistent QFT+, pre-and post-reverters and non-converters. Number of participants in each group, confirmatory statistical analysis and p-value calculations is as in **Figure 2C**.

Results described above suggest that QFT reversion was not associated with a decrease in functional activity nor a change in T cell memory subsets. Since we and others have shown that T cell activation may correlate with M.tb bacterial burden (29, 31, 36), we determined whether QFT reversion was associated with a decrease in T cell activation. Overall, no difference in proportions of activated (HLA-DR+) M.tb-specific (Th1 cytokine+) CD4 T cells, regardless of stimulation, were detected pre- and post-reversion, as well as compared to persistent QFT+ individual and non-converters (**Figure 4**).

### QFT Reversion Is Not Associated With Modulation of Innate, DURT, and B Cell Responses

Since multiple cell types can contribute to IFN-γ production in response to mycobacteria, including NK and DURT cells, and innate cells can also modulate IFN-γ expression in classical T cells, we further explored the potential role of these cells in QFT reversion.

To confirm the capability of innate, DURT and B cell subsets to produce IFN-γ+ in response to *M.tb*, we utilized tSNE analysis (**Supplementary Figure 11**) (46, 47). NK cells, classical CD3+ T cells and MAIT cells were the main contributors to lymphocyte IFN-γ expression in response to M.tb lysate stimulation (**Supplementary Figures 11A, B**). As expected, spontaneous IFN-γ expression in unstimulated samples was mostly detected in NK cells, some T cell subsets and, surprisingly, B cells. NK and γδ T cells were the two major IFN-γ producers in response to *E. coli*.

**Figure 5:**
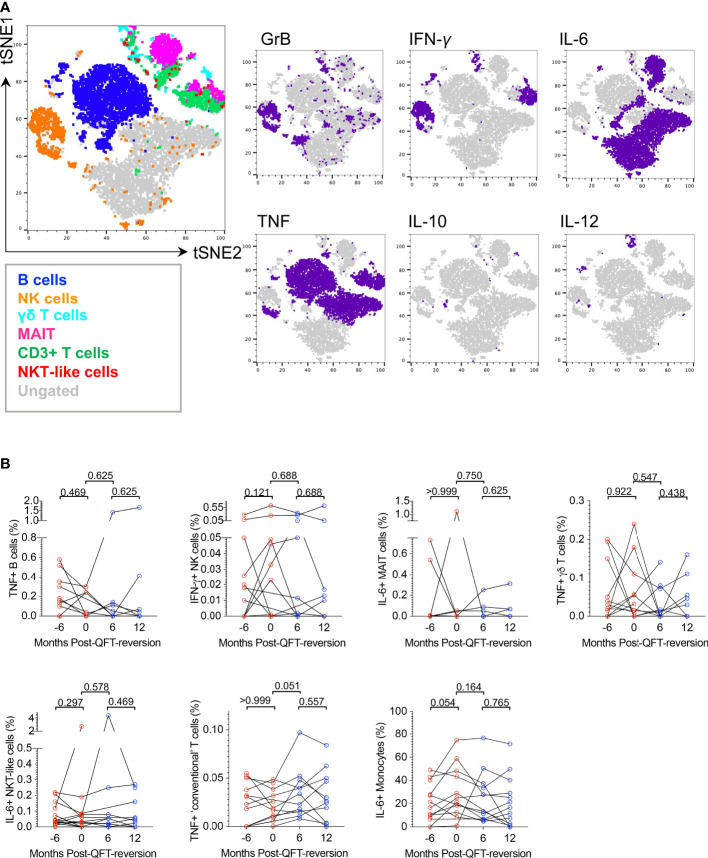
M.tb lysate-reactive innate, DURT and B cell responses do not change upon QFT reversion. **(A)** tSNE visualization of M.tb-reactive lymphocyte cell subsets expressing any cytokine (Granzyme B (GrB), IL-6, IL-10, IL-12, IFN-γ, TNF) in QFT reverters (all visits included). **(B)** Background-subtracted frequencies of M.tb-reactive TNF+ B cells, IFN-γ+ NK cells, IL-6+ MAIT cells, TNF+ γδ T cells, IL-6+ NKT-like cells, TNF+ T cells and IL-6+ monocytes detected pre- and post-reversion. Month -6: n = 13 (QFT+, red symbol), Month 0: n = 11 (QFT+, red symbol), Month 6: n = 11 (QFT-, blue symbol) and Month 12: n = 11 (QFT-, blue symbol). P values were calculated by Wilcoxon signed rank test and p < 0.017 were considered significant.

We further used tSNE analysis to visualize which other cytokines innate, DURT and B cell subsets produce in response to M.tb lysate in all participants combined (**Supplementary Figure 12**) or reverters only (**Figure 5A**). Granzyme B in combination with IFN-γ and/or TNF was mostly expressed by NK cells; IFN-γ was expressed by NK and most T cell subsets; IL-6 was expressed by MAIT, some B cells and “ungated” cells (probably CD14- myeloid cells); TNF was expressed by NK cells, MAIT, classical T cells and B cells. The expression of IL-10 and IL-12 was very low and variable in all cell types in response to M.tb stimulation.

To compare innate, DURT and B cell responses longitudinally in reverters and cross-sectionally across study groups, frequencies of cytokine+ cells in unstimulated samples were subtracted from those in the M.tb lysate-stimulated samples. For cell types expressing multiple functional markers, co-expression profiles were also evaluated (data not shown). We found no statistical differences in innate, DURT and B cell responses over time in reverters, nor when reverters were compared with persistent QFT+ and non-converters (**Figure 5B** and data not shown). Except for monocytes, responses were generally low and variable, and unfortunately only half the number of participants in each group could be included in this analysis.

Overall, we confirmed that M.tb lysate induces cytokine production by innate, DURT and B cells, however, these functional responses did not differ upon QFT reversion or across study groups in the small subset of participants included in this analysis.

### QFT Reverters Are More Similar to Non-Converters Than Persistent QFT+ Individuals

**Figure 6:**
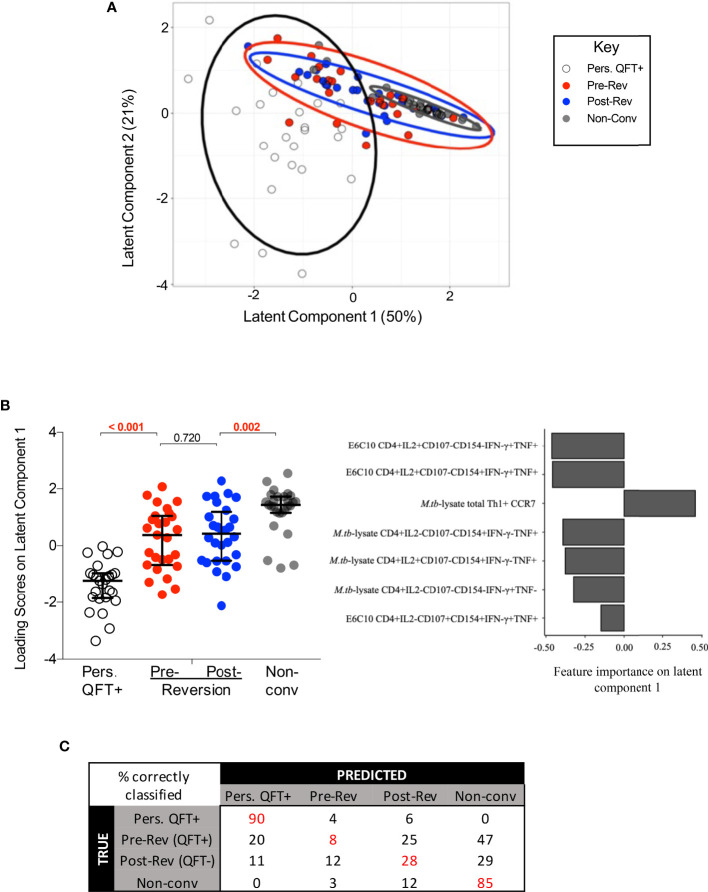
Integrated analysis of classical T cell, innate, DURT and B cell responses to M.tb. **(A)** The PLS-DA biplot shows the first and second latent components from the model built on LASSO-selected features. Each dot represents a participant, all participants were included (n = 30 per group). The thick line curves denote the 95% confidence ellipses for each group. **(B)** Left: loading scores of each group on latent component one, where the pre- and post- reverter groups were compared using Wilcoxon’s signed rank paired test and the other groups were compared using Mann Whitney’s U test. Right: bar graph indicating feature importance in latent component one. **(C)** The average confusion matrix across 1000 bootstrapped samples after a cross-validation of the PLS-DA model. Each row represents the true outcome in the testing set and each column the outcome predicted by the model. Values in red represent the % of correctly classified observations.

To further explore the relationship between QFT reverters and the control groups, the two (adaptive and innate) datasets were integrated and fitted to a PLS-DA model using LASSO selected features (**Figure 6**). The feature selection was based on persistent QFT+ and non-converters, then the model was applied to the reverters to evaluate whether they constituted a distinct population from the control groups. The LASSO model, blinded to the QFT reverters, identified seven stratifying features (all from the adaptive dataset) that could best distinguish persistent QFT+ and non-converters (**Figure 6B**, right). Using these seven features as exploratory variables and the four groups (persistent QFT+, pre-reverters, post-reverters and non-converters) as a response, a PLS-DA model was built to the vast scaled and MFA-imputed integrated dataset. In two dimensions, the model captured 71% of the total variability and was able to successfully separate persistent QFT+ and non-converter observations. The pre- and post-reversion groups greatly overlapped with each other and were positioned in between the controls (**Figure 6A**). The reverters, regardless of QFT status, had intermediate loading scores on latent component one, which were significantly different from both control groups (**Figure 6B**, left). The persistent QFT positives loaded negatively on the first component and the top variables that drove this were two CFP-10/ESAT-6-specific IFN-γ+ CD4+ T cell subsets. The non-converters, on the other hand, loaded positively on the first component and the expression of CCR7 on M.tb-lysate-specific total Th1 cells drove this separation (**Figure 6B**, right). The second latent component, which captured 21% of the total variability, could successfully separate the persistent QFT positives from the other three groups, but failed to differentiate the non-converters from the QFT reverters (**Supplementary Figure 13A**). The main features distinguishing reverters and non-converters from persistent QFT+ were M.tb lysate-specific IFN-γ- CD4 T cells (**Supplementary Figure 13B**).

The performance of this PLS-DA model was then assessed *via* a cross-validation procedure and the average misclassification error after 1000 bootstrapped iterations was 0.48. The misclassification rate of persistent QFT positives and non-converters were 10% and 15%, respectively, while the misclassification rates of pre- and post-reverters were 92% and 72%, respectively. The average confusion matrix across the bootstrapped samples (**Figure 6C**) showed that about half of the observations from both reverter groups were misclassified as non-converters, which is twice as many as those that were classified as persistent QFT positives, or correctly classified as reverters.

Since reverters were not separated according to QFT status, a second PLS-DA model was built to assess whether the reverters could be stratified according to CFP-10/ESAT-6 responder status defined by MIMOSA (40). Responder and non-responder reverters were still not significantly different from each other and were significantly different from the control cohorts (**Supplementary Figure 13C**).

Finally, a LASSO model was built including all 3 groups (i.e. QFT reverters, persistent positives and negatives) as a class outcome and similar variables to the LASSO model that was blinded to the reverters were identified. Consequently, the performance remained the same, with the reverters frequently misclassified (data not shown).

Taken together, statistical modelling performed on all available data confirmed that innate, DURT and B cell responses did not contribute to the stratification of the study groups, and that M.tb-specific CD4 T cell responses in reverters were overall more similar to those detected in non-converters compared to persistent QFT+.

## Discussion

Based on TST reversion studies in humans and guinea pig animal models, researchers hypothesize that QFT and/or TST reversion is associated with M.tb clearance or a reduction of bacterial burden (7). We found that QFT reversion was observed in a heterogenous group of individuals. In our cohort, none of the classical T cell, innate, DURT and B cell features we measured changed upon QFT reversion, suggesting that at least in some individuals reversion is due to QFT variability. However, reverters consistently displayed intermediate magnitude and differentiation status of M.tb-specific CD4 T cell responses compared to non-converters and persistent QFT+ individuals. Overall, reverters shared more similar features with non-converters compared to persistent QFT+ individuals, suggesting that reverters are generally exposed to very low or no antigen *in vivo*, which is consistent with well-controlled or previously cleared M.tb infection.

In our cohort of QFT reverters, functional and memory M.tb-specific CD4 T cell profiles did not change upon QFT reversion in response to a variety of M.tb immunodominant antigens. However, less than half of the reverters were classified as CD4 T cell responders to CFP-10/ESAT-6 stimulation both before and after reversion. This highlights the heterogeneity of QFT reverters, which include individuals who have low M.tb-specific immune responses which fluctuate around the QFT cut-off, as well as individuals who have low but detectable M.tb-specific responses despite falling below the QFT cut-off. M.tb-specific cytokine-producing CD4 T cells detected in reverters were of intermediate magnitude that fell between those observed in non-converters and persistent QFT+. These results are not consistent with findings by Jenum and colleagues, who reported that magnitude and cytokine co-expression profile of M.tb-specific Th1 CD4 T cells were not significantly different between IGRA reverters (n=6) and IGRA+ individuals [n=14, (9)]. M.tb-specific T cell activation (HLA-DR expression), which is likely associated with *in vivo* bacterial load during recent infection and active tuberculosis disease (29, 31, 32, 36), was very low and not significantly different across study groups. Based on these results, we found no clear evidence against the *reversion = clearance* hypothesis. In fact, the low level of activation in all groups suggests that even persistently QFT+ individuals are exposed to low bacterial burden *in vivo*, which does not trigger higher T cell activation than M.tb-uninfected individuals (non-converters).

We detected higher levels of CFP-10/ESAT-6-specific T_TM_ and early-differentiated IFN-γ-TNF+IL-2± memory subsets, but lower levels of IFN-γ+TNF+ T_E_ cells and functional differentiation in reverters compared with persistent QFT+. Further, with the exception of lower proportions of M.tb lysate-specific T_SCM_, memory/functional subsets were not significantly different between reverters and non-converters. These findings suggest that reverters likely have a controlled, low-grade M.tb infection, or may have even had cleared infection, prior to detection of the QFT reversion. These results also highlight that once M.tb infection is established, immunological memory, consisting of both effector and T_SCM_/T_CM_ cell subsets, is sustained even upon QFT reversion.

We hypothesized that distinct innate, DURT and B cell responses could contribute to controlling M.tb infection or modulate IFN-γ production resulting in QFT reversion. Overall, we observed no significant differences in these immune responses to M.tb lysate stimulation pre- and post-QFT-reversion for any of the functional molecules (IFN-γ, IL-6, TNF, IL-10, IL-12 and Granzyme B) expressed by different cell types (including monocytes, B, NK and DURT cells), nor differences between the groups. Unfortunately, this analysis was conducted on a small subset of participants, and therefore it was likely underpowered to detect small differences.

To evaluate whether a combination of features from the adaptive T cell as well as the innate, DURT and B cell datasets could better capture changes in the reverters, or classify them as distinct from persistent QFT+ and non-converters, we integrated and modelled all the outcomes generated in separate experiments. Performing LASSO feature selection prior to fitting the PLS-DA model improved the computational efficiency of the model and was used to identify the most differentiating features in the dataset that could discriminate between persistent QFT+ and non-converters. The LASSO model, and hence the PLS-DA model, were therefore blinded to the relationship between the reverters and the control cohorts. The model did not distinguish between QFT+ and QFT- reverters (*i.e.* pre- and post-reversion), nor between reverters who did or did not respond to CFP-10/ESAT-6 antigen stimulation, thereby confirming that overall there were no distinct immunological features that changed upon reversion. However, the model did not consistently identify the reverters as either one of the control cohorts, suggesting that the reverters are a biologically distinct but heterogeneous group of individuals with intermediate features between QFT+ and QFT- control groups. The initial PLS-DA model built to the integrated dataset was validated *via* a cross-validation procedure and yielded a poor average performance, with pre- and post-reverter observations that were more often misclassified than correctly classified. Specifically, the majority of the observations in the pre- and post-reverter groups were misclassified as non-converters more than they were correctly classified as reverters or misclassified as persistent QFT positives. This suggests that immune features measured in reverters are more similar to non-converters than persistent QFT positives, implying that the QFT reverters may have cleared, or at least controlled *M.tb* infection to a very low load. However, given the small sample size of the dataset, using the misclassification error rate is a misleading performance metric and is difficult to draw definitive conclusions.

We acknowledge that this study has several other limitations, including discordance between QFT and TST responses in reverters; the majority of reverters were persistently TST positive during follow-up. This result suggests that TST may be a more sensitive test to detect less immunodominant or low mycobacteria-reactive responses than QFT. Purified protein derivative administered for TST consists of hundreds of antigens, in addition to the two antigens used in QFT. In addition, the long time frame (48-72 hours) between purified protein derivative injection and measurement of induration allows recruitment of lymphoid tissue-resident memory cells and for early differentiated cells to proliferate and induce a delayed-type hypersensitivity reaction, while QFT is a shorter *ex vivo* assay that detects circulating effector IFN-γ+ T cells. Our finding that early differentiated M.tb-specific CD4 T cells persisted in reverters is therefore consistent with their sustained TST reactivity even upon QFT reversion. Due to the limited amount of samples available, we could not perform long-term cell stimulations, which may have further confirmed higher proportions of early differentiated M.tb-specific CD4 T cells in reverters compared to persistent QFT positives (48).

We may have observed different M.tb-specific memory and functional kinetics if we had selected participants with concordant QFT and TST reversion. Unfortunately, not enough individuals with this phenotype in our larger adolescent cohort study had sufficient PBMC available.

We measured M.tb-specific responses to CFP-10/ESAT-6, other M.tb-specific immunodominant antigens EspC/EspF/Rv2348 (49); and to M.tb lysate, which includes antigens cross-reactive with BCG and environmental mycobacteria. We cannot exclude that T cell responses to other M.tb antigens expressed at varying abundance by M.tb bacilli with different metabolic states, such as the Ag85 complex or latency-associated proteins (50, 51), may be associated with reversion.

We did not measure expression of other cytokines or functions known to contribute to M.tb control, such as IL-17, which might be associated with protective immunity in non-human primates (46, 47). We did not include IL-17 in our panel because mycobacterial stimulation of human PBMC induces very low IL-17 production by T cells (52), which would have been insufficient to allow phenotyping of these cells.

Finally, we do not know when the cohort of reverters studied here acquired infection, as they were selected to be QFT+ for >6 months before reversion. Whether immune features associated with transient infection (QFT conversion followed by rapid reversion) or potential clearance of established infection (sustained remote QFT conversion followed by reversion) share similarities remains unknown.

We propose that QFT reverters represent a heterogenous grouping of individuals in the tuberculosis spectrum. This grouping includes individuals with detectable, pre-reversion levels of IFN-γ in the QFT assay, which are not robustly and universally detectable at a single cell level by flow cytometry, as well as post-reversion QFT- individuals with early differentiated T cell responses detected by flow cytometry and TST. In some individuals QFT reversion may therefore be simply due to technical variability of the assay. In other individuals, the low magnitude of M.tb-specific CD4 T cells responses and the memory and functional profiles observed in QFT reverters shared more characteristics with non-converters than persistently infected (QFT+) individuals, which is consistent with low or no *in vivo* antigen exposure and may indicate controlled or cleared M.tb infection.

## Data Availability Statement

The datasets presented in this study is accessible on ZivaHub (https://doi.org/10.25375/uct.14635503.v1).

## Ethics Statement

The studies involving human participants were reviewed and approved by the University of Cape Town Human Research Ethics Committee, protocol references: 045/2005, 102/2017. Written informed consent to participate in this study was provided by the participants’ legal guardian/next of kin.

## Author Contributions

EN, MH, and TS raised funding. EN, MH, TS, JA, and MR designed the study. CM, PS, CS, BM, TR, and NB generated the data. CM, PS, TL, and VR analyzed the data. FL supervised data analysis. CM, PS, TL, EN, TS, and FL interpreted the results. CM, PS, TL, and EN wrote the manuscript. All authors contributed to the article and approved the submitted version.

## Funding

US NIH (R21AI127121 and BAA-NIAID-NIHAI201700104) funded the study. This work was also supported by Global Health Grant OPP1066265 from the Bill & Melinda Gates Foundation. The ACS study was supported by Aeras and BMGF GC12 (grant 37885) for QFT testing. The South African National Research Foundation and the University of Cape Town funded scholarships to CM. PS received a fellowship from the Wellcome Centre for Infectious Diseases Research (CIDRI) in Africa. TL received a bursary from the National Research Foundation (NRF) of South Africa.

## Conflict of Interest

The authors declare that the research was conducted in the absence of any commercial or financial relationships that could be construed as a potential conflict of interest.

## Publisher’s Note

All claims expressed in this article are solely those of the authors and do not necessarily represent those of their affiliated organizations, or those of the publisher, the editors and the reviewers. Any product that may be evaluated in this article, or claim that may be made by its manufacturer, is not guaranteed or endorsed by the publisher.

## LIST OF ACS STUDY TEAM MEMBERS

ACS study team members: Hassan Mahomed, Willem A. Hanekom, Fazlin Kafaar, Leslie Workman, Humphrey Mulenga, Rodney Ehrlich, Mzwandile Erasmus, Deborah Abrahams, Anthony Hawkridge, E. Jane Hughes, Sizulu Moyo, Sebastian Gelderbloem, Ashley Veldsman, Michele Tameris, Hennie Geldenhuys, Gregory Hussey.
