# Supplementary material for: Mycobacterium tuberculosis-Specific T Cell Functional, Memory, and Activation Profiles in QuantiFERON-Reverters Are Consistent With Controlled Infection
Source: Front Immunol. 2021 Aug 30;12:712480. doi: 10.3389/fimmu.2021.712480 (PMC8435731; doi:10.3389/fimmu.2021.712480)
Supplement: Supplementary file 1 [file DataSheet_1.zip › Supp Figure 10.pdf]

Supplementary Figure 10: Reverters have lower CFP-10/ESAT-6-specific functional differentiation than persistent QFT+ individuals.

A

Stim	IFN-γ	TNF	IL-2	Median relative proportion of Th1 Cyt+ cells (%): Pers. QFT+	Pre-Reversion	Post-Reversion	Non-Conv	P values: Pers. QFT+ vs Pre-Rev	Pre- vs Post-Rev	Post-Rev vs Non-Conv
M.tb-Lysate	-	-	+	1.2	2.0	2.5	4.2	**0.013**	0.614	0.03
	-	+	-	10.8	11.0	10.7	26.1	0.097	0.104	**0.003**
	-	+	+	6.0	9.9	7.1	7.6	0.044	**0.013**	0.849
	+	+	+	37.3	38.0	33.7	20.1	0.65	0.514	0.021
	+	+	-	29.1	24.5	26.8	19.7	0.198	0.131	0.02
	+	-	+	1.7	1.2	1.9	1.4	0.146	**0.012**	0.406
	+	-	-	9.0	6.1	7.9	9.2	0.036	0.076	0.912
CFP-10/ESAT-6	-	-	+	3.3	5.4	6.2		0.156	0.683	
	-	+	-	17.1	35.1	25.0		**< 0.001**	0.067	
	-	+	+	7.4	20.5	19.1		**0.003**	0.76	
	+	+	+	35.8	11.3	17.6		**0.003**	0.359	
	+	+	-	19.4	9.3	13.4		**< 0.001**	0.025	
	+	-	+	2.4	1.9	1.3		0.255	0.919	
	+	-	-	8.9	3.7	5.8		0.020	0.185	
EspC/EspF/Rv2348	-	-	+	2.3						
	-	+	-	9.6						
	-	+	+	4.4						
	+	+	+	29.8						
	+	+	-	27.0						
	+	-	+	2.2						
	+	-	-	7.2						

Scale: Median relative proportion of Th1 Cyt+ cells (%) — 0, 10, 20, 30, 40

B

Stim	RA	R7	27	G1	Popn	Median relative proportion of Th1 Cyt+ cells (%): Pers. QFT+	Pre-Reversion	Post-Reversion	Non-Conv	P values: Pers. QFT+ vs Pre-Rev	Pre- vs Post-Rev	Post-Rev vs Non-Conv
M.tb-Lysate	+	+	+	-	T_{SCM}	2.1	2.1	1.7	9.8	0.279	0.401	**0.001**
	-	+	+	-	T_{CM}	8.2	9.4	8.3	12.5	0.313	0.185	0.023
	-	-	+	-	T_{TM}	29.5	31.0	28.4	25.9	0.788	0.695	0.177
	-	-	+	+	T_{TM}	10.6	8.9	10.7	6.3	0.69	0.121	0.201
	-	-	-	-	T_E	23.0	24.9	21.2	18.6	0.598	0.271	0.787
	-	-	-	+	T_E	18.9	13.3	13.9	8.5	0.146	0.032	0.131
CFP-10/ESAT-6	+	+	+	-	T_{SCM}	4.8	5.0	6.0		0.743	0.683	
	-	+	+	-	T_{CM}	7.8	16.3	12.1		**0.004**	0.541	
	-	-	+	-	T_{TM}	38.5	36.9	35.0		0.543	0.61	
	-	-	+	+	T_{TM}	9.1	4.8	5.6		0.326	1	
	-	-	-	-	T_E	18.4	20.5	17.4		0.289	1	
	-	-	-	+	T_E	8.5	5.4	7.1		0.185	0.154	
EspC/EspF/Rv2348	+	+	+	-	T_{SCM}	4.3						
	-	+	+	-	T_{CM}	5.7						
	-	-	+	-	T_{TM}	30.4						
	-	-	+	+	T_{TM}	4.7						
	-	-	-	-	T_E	29.6						
	-	-	-	+	T_E	9.6						

Scale: Median % of Th1 Cyt+ cells — 0, 10, 20, 30, 40
