# Supplementary material for: Mycobacterium tuberculosis-Specific T Cell Functional, Memory, and Activation Profiles in QuantiFERON-Reverters Are Consistent With Controlled Infection
Source: Front Immunol. 2021 Aug 30;12:712480. doi: 10.3389/fimmu.2021.712480 (PMC8435731; doi:10.3389/fimmu.2021.712480)

Supplementary Figure 12: tSNE analysis of *M.tb* lysate stimulated lymphocytes expressing any cytokine.

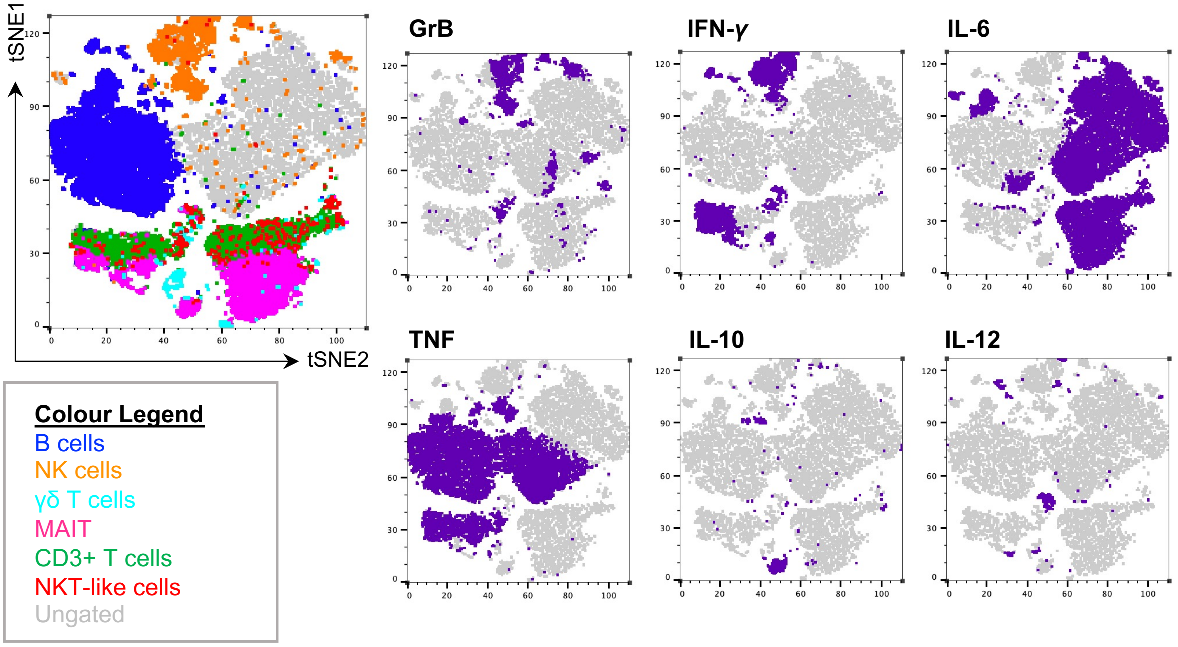

Supplement: Supplementary file 1 [file DataSheet_1.zip › Supp Figure 12.pdf]
