# Supplementary material for: Mycobacterium tuberculosis-Specific T Cell Functional, Memory, and Activation Profiles in QuantiFERON-Reverters Are Consistent With Controlled Infection
Source: Front Immunol. 2021 Aug 30;12:712480. doi: 10.3389/fimmu.2021.712480 (PMC8435731; doi:10.3389/fimmu.2021.712480)
Supplement: Supplementary file 1 [file DataSheet_1.zip › Supp Figure 4.pdf]

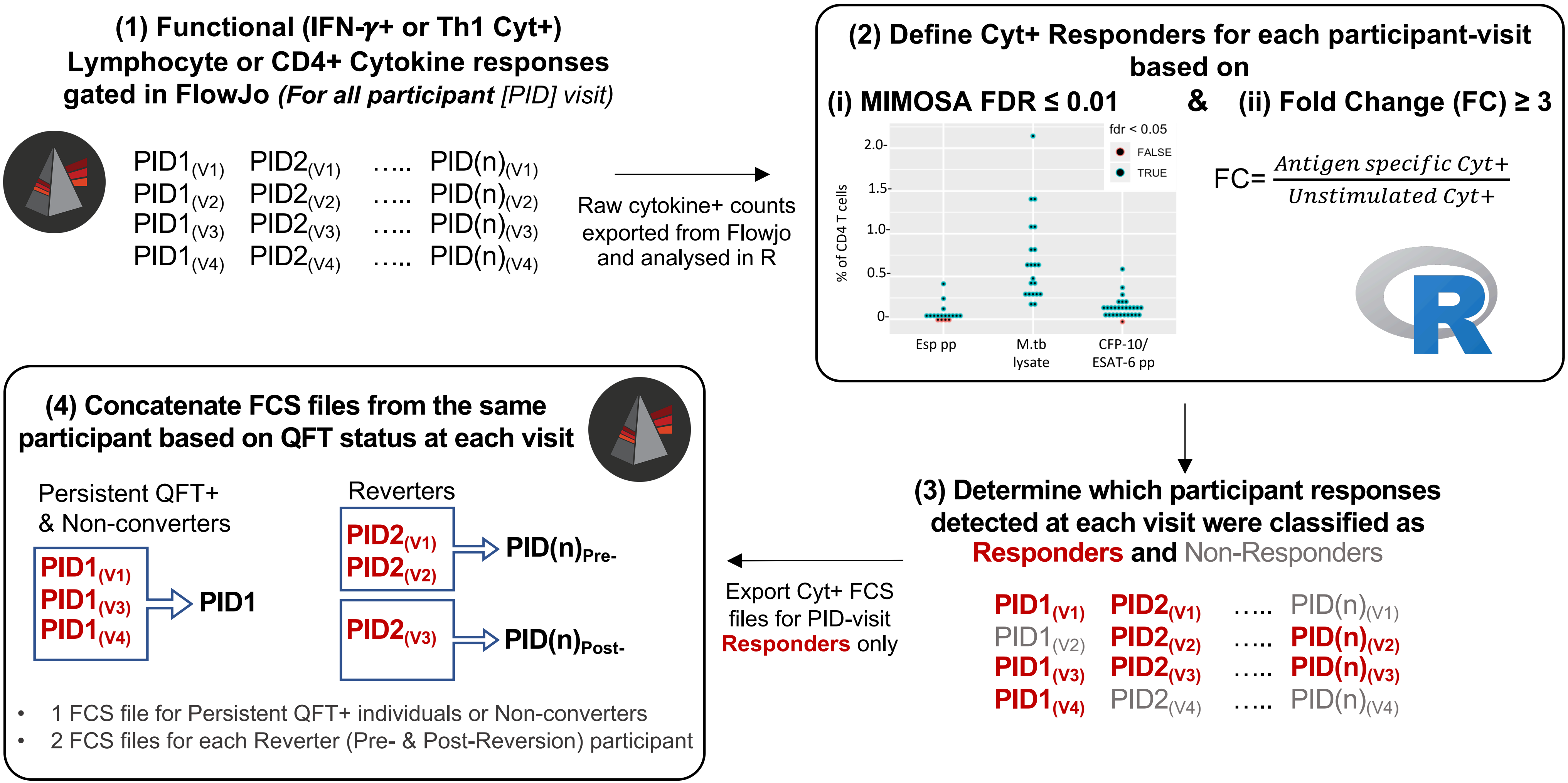

(1) Functional (IFN-γ+ or Th1 Cyt+) Lymphocyte or CD4+ Cytokine responses gated in FlowJo (For all participant [PID] visit)
PID1(V1) PID2(V1) ….. PID(n)(V1)
PID1(V2) PID2(V2) ….. PID(n)(V2)
PID1(V3) PID2(V3) ….. PID(n)(V3)
PID1(V4) PID2(V4) ….. PID(n)(V4)
Raw cytokine+ counts exported from Flowjo and analysed in R
(2) Define Cyt+ Responders for each participant-visit based on
(i) MIMOSA FDR ≤ 0.01 & (ii) Fold Change (FC) ≥ 3
fdr < 0.05
FALSE
TRUE
% of CD4 T cells
2.0-
1.5-
1.0-
0.5-
0-
Esp pp
M.tb lysate
CFP-10/ ESAT-6 pp
FC= Antigen specific Cyt+ / Unstimulated Cyt+
(3) Determine which participant responses detected at each visit were classified as Responders and Non-Responders
PID1(V1) PID2(V1) ….. PID(n)(V1)
PID1(V2) PID2(V2) ….. PID(n)(V2)
PID1(V3) PID2(V3) ….. PID(n)(V3)
PID1(V4) PID2(V4) ….. PID(n)(V4)
Export Cyt+ FCS files for PID-visit Responders only
(4) Concatenate FCS files from the same participant based on QFT status at each visit
Persistent QFT+ & Non-converters
PID1(V1)
PID1(V3)
PID1(V4)
PID1
Reverters
PID2(V1)
PID2(V2)
PID(n)Pre-
PID2(V3)
PID(n)Post-
• 1 FCS file for Persistent QFT+ individuals or Non-converters
• 2 FCS files for each Reverter (Pre- & Post-Reversion) participant
