# Supplementary material for: Mycobacterium tuberculosis-Specific T Cell Functional, Memory, and Activation Profiles in QuantiFERON-Reverters Are Consistent With Controlled Infection
Source: Front Immunol. 2021 Aug 30;12:712480. doi: 10.3389/fimmu.2021.712480 (PMC8435731; doi:10.3389/fimmu.2021.712480)
Supplement: Supplementary file 1 [file DataSheet_1.zip › Supp Figure 5.pdf]

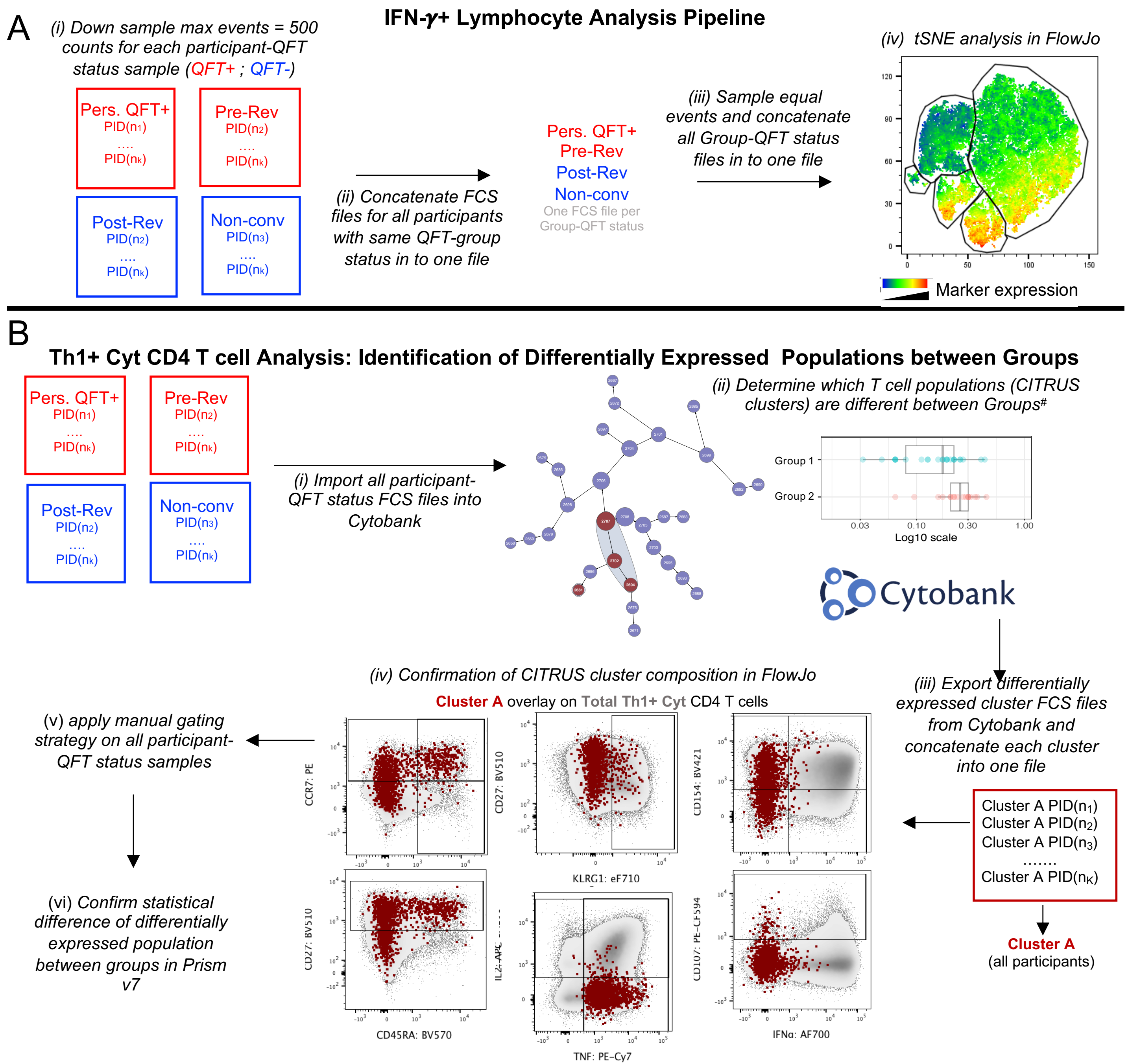

A
IFN-γ+ Lymphocyte Analysis Pipeline
(i) Down sample max events = 500 counts for each participant-QFT status sample (QFT+ ; QFT-)
Pers. QFT+
PID(n1)
....
PID(nk)
Pre-Rev
PID(n2)
....
PID(nk)
Post-Rev
PID(n2)
....
PID(nk)
Non-conv
PID(n3)
....
PID(nk)
(ii) Concatenate FCS files for all participants with same QFT-group status in to one file
Pers. QFT+
Pre-Rev
Post-Rev
Non-conv
One FCS file per Group-QFT status
(iii) Sample equal events and concatenate all Group-QFT status files in to one file
(iv) tSNE analysis in FlowJo
Marker expression
B
Th1+ Cyt CD4 T cell Analysis: Identification of Differentially Expressed Populations between Groups
Pers. QFT+
PID(n1)
....
PID(nk)
Pre-Rev
PID(n2)
....
PID(nk)
Post-Rev
PID(n2)
....
PID(nk)
Non-conv
PID(n3)
....
PID(nk)
(i) Import all participant-QFT status FCS files into Cytobank
(ii) Determine which T cell populations (CITRUS clusters) are different between Groups#
Group 1
Group 2
Log10 scale
Cytobank
(iii) Export differentially expressed cluster FCS files from Cytobank and concatenate each cluster into one file
Cluster A PID(n1)
Cluster A PID(n2)
Cluster A PID(n3)
....
Cluster A PID(nK)
Cluster A
(all participants)
(iv) Confirmation of CITRUS cluster composition in FlowJo
Cluster A overlay on Total Th1+ Cyt CD4 T cells
CCR7: PE
CD27: BV510
CD154: BV421
KLRG1: eF710
CD45RA: BV570
IL2: APC
CD107: PE-CF594
TNF: PE-Cy7
IFNα: AF700
(v) apply manual gating strategy on all participant-QFT status samples
(vi) Confirm statistical difference of differentially expressed population between groups in Prism v7
