# Supplementary material for: Mycobacterium tuberculosis-Specific T Cell Functional, Memory, and Activation Profiles in QuantiFERON-Reverters Are Consistent With Controlled Infection
Source: Front Immunol. 2021 Aug 30;12:712480. doi: 10.3389/fimmu.2021.712480 (PMC8435731; doi:10.3389/fimmu.2021.712480)

A

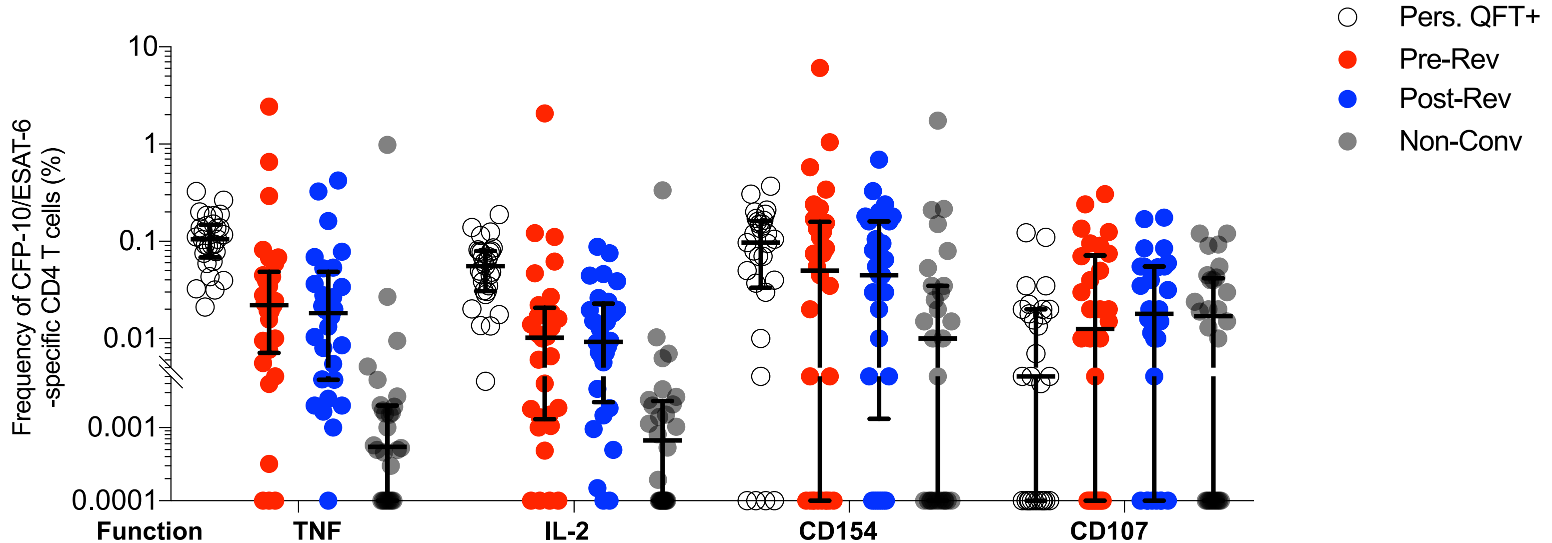

Function		TNF	IL-2	CD154	CD107
P-value	Pers. QFT**+** vs Pre-Rev	**0.001**	**<0.001**	0.289	0.159
	Pre- vs Post-Rev	0.036	0.711	0.648	0.541
	Post-Rev vs Non-conv	**<0.001**	**0.001**	0.084	0.515

B

	Function co-expression profile					Groups				P-value		
	CD154	IL-2	TNF	IFN-γ	CD107	Pers. QFT+	Pre-Rev	Post-Rev	Non-conv	Pers. QFT**+** vs Pre-Rev	Pre- vs Post-Rev	Post-Rev vs Non-Conv
Early Differentiation	+	-	-	-	-	0.0148	0.0492	0.0305	0.0084	0.244	0.959	0.065
	-	-	-	-	+	0.0050	0.0164	0.0246	0.0163	0.129	0.955	0.302
	+	-	+	-	-	0.0077	0.0029	0.0037	0.0003	0.190	0.168	**< 0.001**
	-	-	+	-	-	0.0061	0.0022	0.0023	0.0010	0.056	0.489	**0.024**
	+	+	+	-	-	0.0058	0.0024	0.0023	0.0000	0.101	0.622	**< 0.001**
	-	+	+	-	-	0.0005	0.0002	0.0001	0.0000	0.162	0.149	**0.031**
Late Differentiation	+	+	+	+	-	0.0292	0.0047	0.0035	0.0000	**< 0.001**	0.089	**< 0.001**
	-	+	+	+	-	0.0078	0.0009	0.0006	0.0000	**< 0.001**	0.063	**< 0.001**
	+	-	+	+	-	0.0159	0.0027	0.0027	0.0000	**< 0.001**	0.936	**< 0.001**
	-	-	+	+	-	0.0145	0.0009	0.0011	0.0001	**< 0.001**	0.793	**0.009**
	+	+	-	+	-	0.0005	0.0003	0.0003	0.0000	0.190	0.402	**0.004**
	-	+	-	+	-	0.0001	0.0000	0.0000	0.0000	**< 0.001**	0.271	0.640
	+	-	-	+	-	0.0017	0.0006	0.0002	0.0000	**0.030**	0.334	0.194
	-	-	-	+	-	0.0065	0.0013	0.0014	0.0006	**< 0.001**	0.846	**0.027**

Scale

Median Freq. of CFP-10/ESAT-6-specific CD4+ T cells (%)

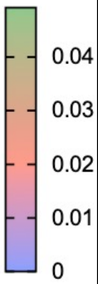

Supplement: Supplementary file 1 [file DataSheet_1.zip › Supp Figure 9.pdf]
