# Supplementary figures and images for: Mycobacterium tuberculosis-Specific T Cell Functional, Memory, and Activation Profiles in QuantiFERON-Reverters Are Consistent With Controlled Infection
Source: Front Immunol. 2021 Aug 30;12:712480. doi: 10.3389/fimmu.2021.712480 (PMC8435731; doi:10.3389/fimmu.2021.712480)

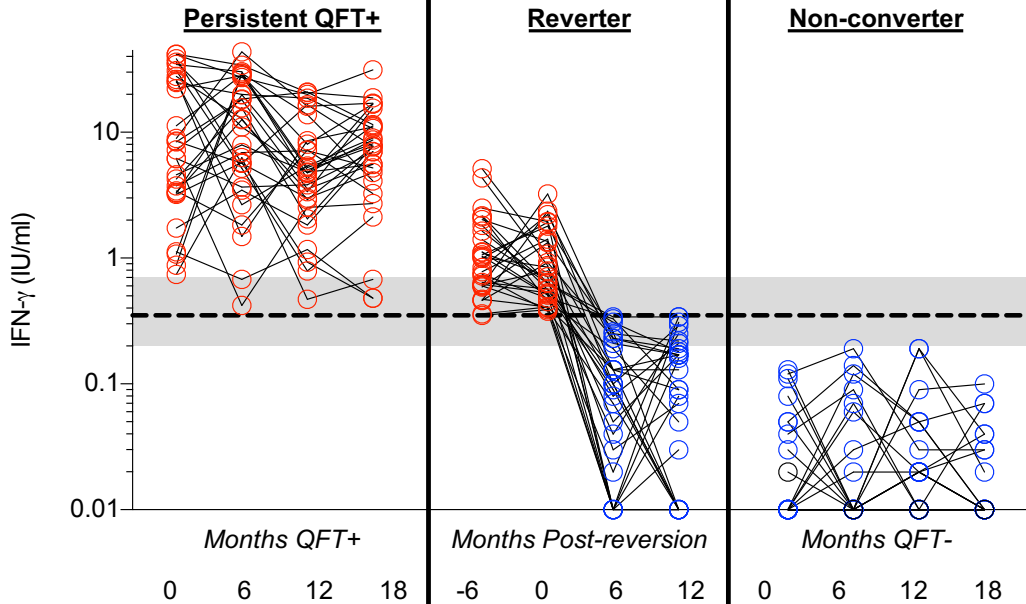

Supplement: Supplementary file 1 [file DataSheet_1.zip › Supp Figure 1.pdf]

**Supplementary Figure 11: IFN- $\gamma$  expressing lymphocytes**

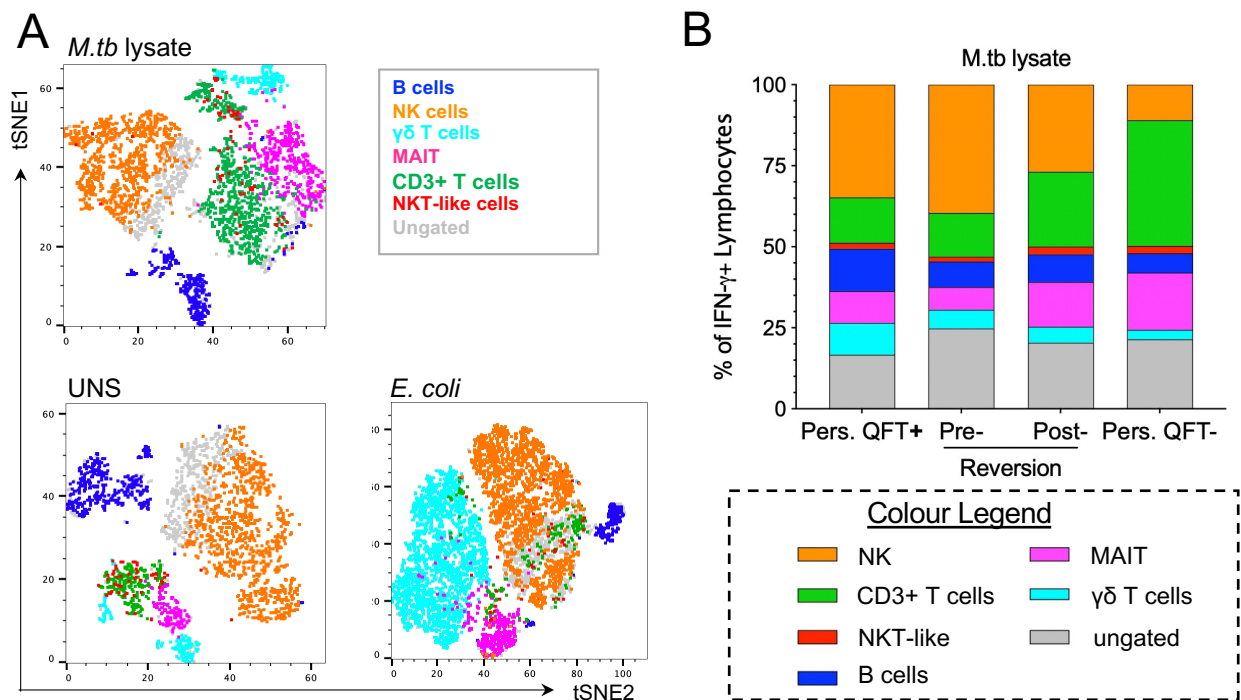

Supplement: Supplementary file 1 [file DataSheet_1.zip › Supp Figure 11.pdf]

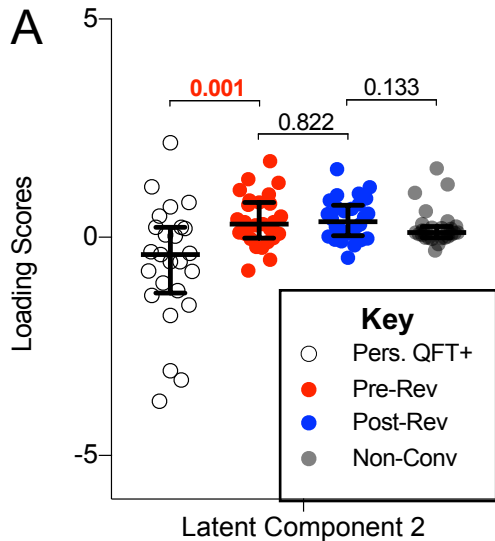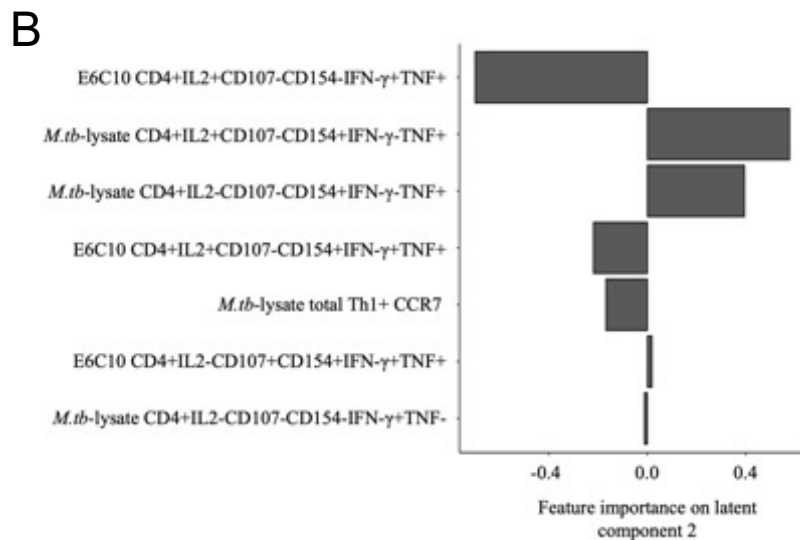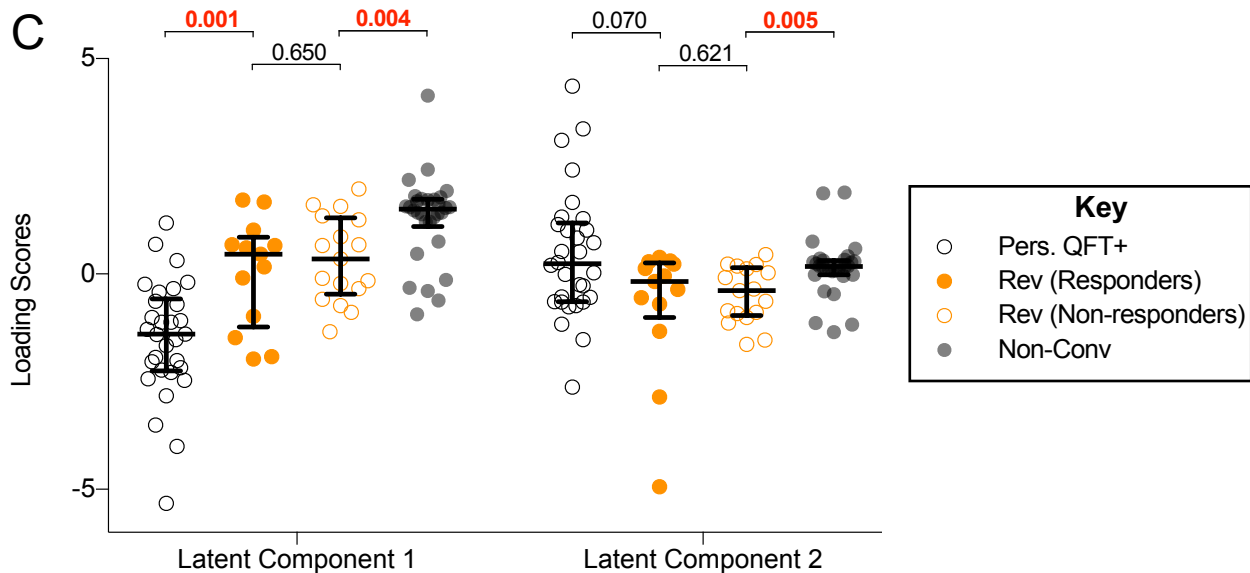

Supplement: Supplementary file 1 [file DataSheet_1.zip › Supp Figure 13.pdf]

## Total Lymphocytes

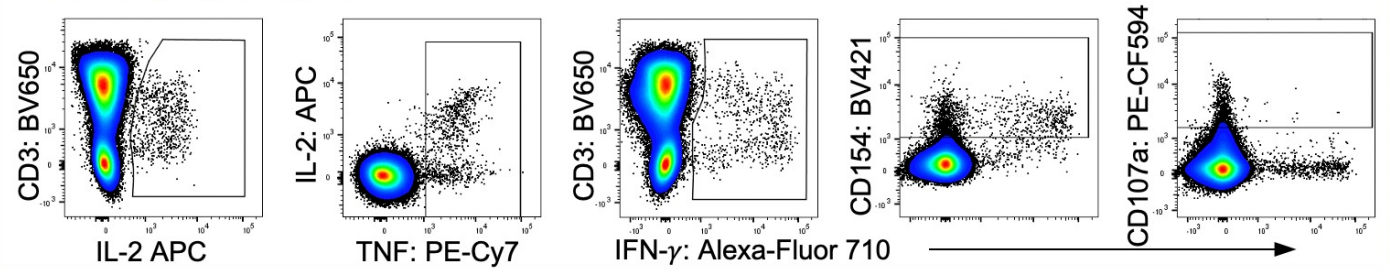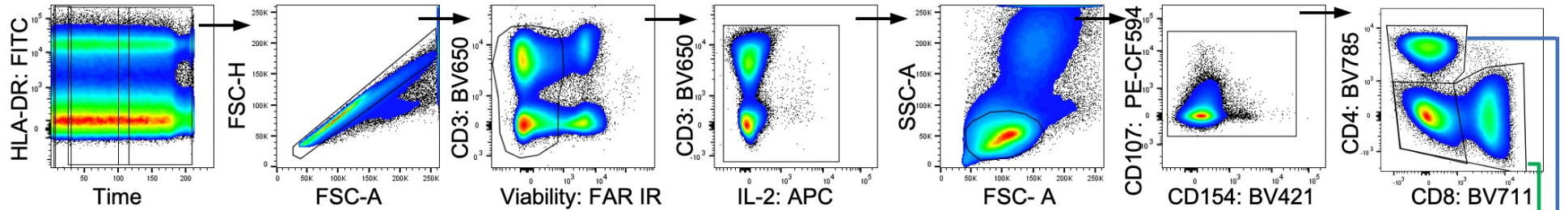

## CD4 T Cells

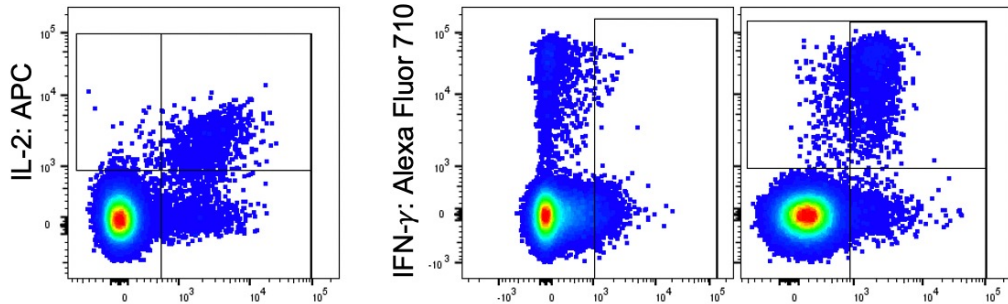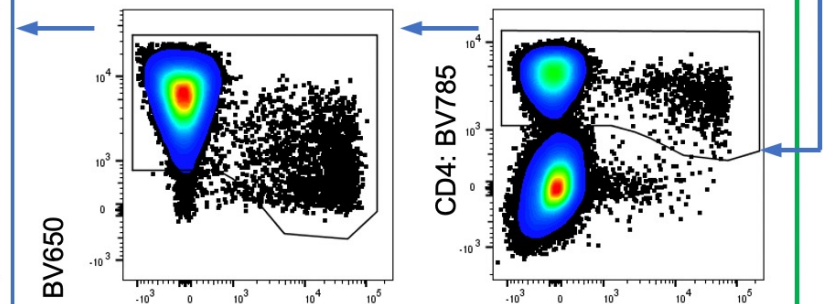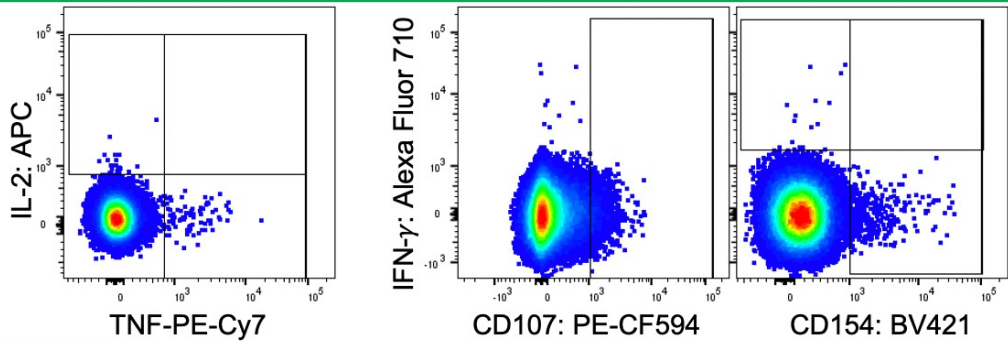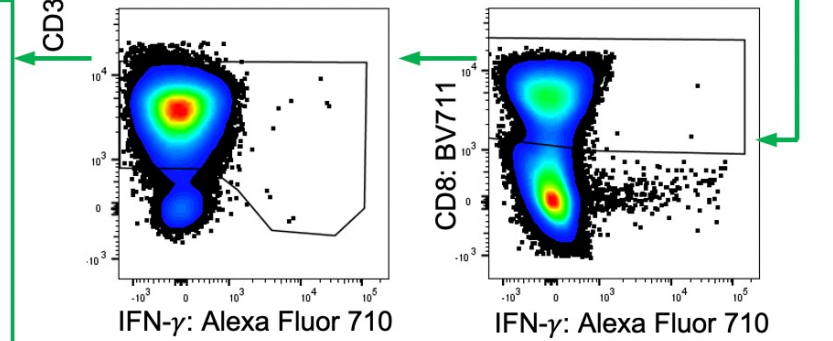

## CD8 T Cells

Supplement: Supplementary file 1 [file DataSheet_1.zip › Supp Figure 2.pdf]

**A**IFN- $\gamma$ + CD4 T cells (%)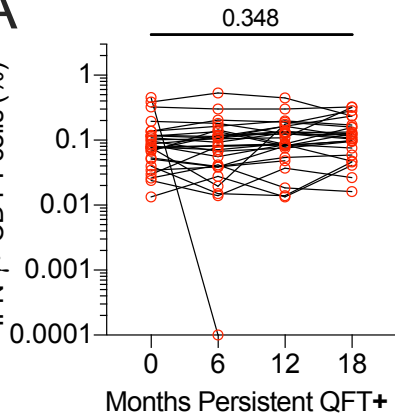**B**IFN- $\gamma$ + CD4 T cells (%)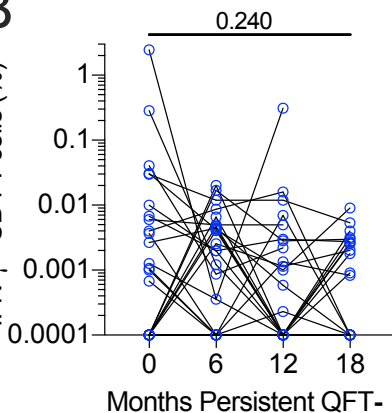**C**IFN- $\gamma$ + CD4 T cells (%)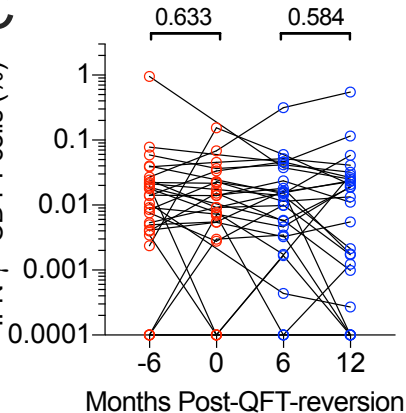

Supplement: Supplementary file 1 [file DataSheet_1.zip › Supp Figure 3.pdf]

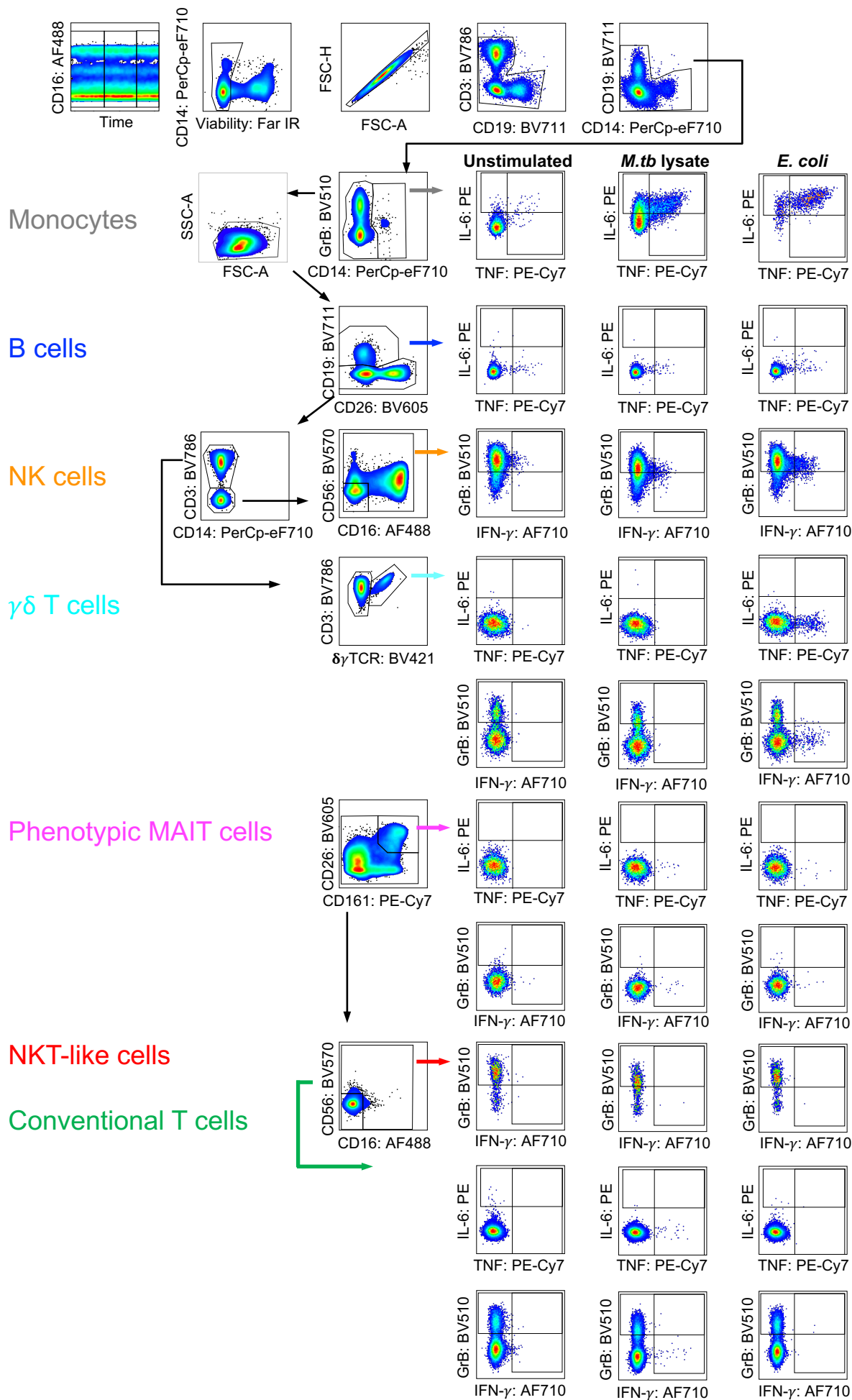

Supplement: Supplementary file 1 [file DataSheet_1.zip › Supp Figure 6.pdf]

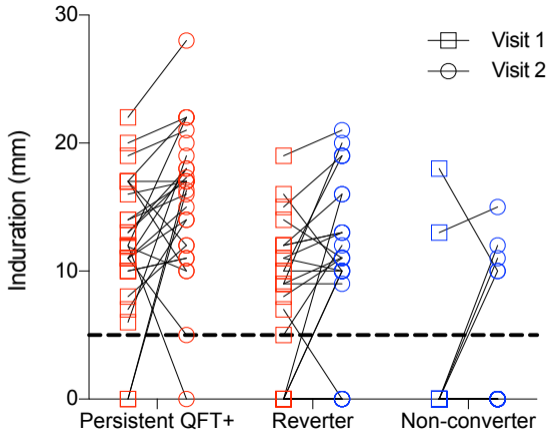

Supplement: Supplementary file 1 [file DataSheet_1.zip › Supp Figure 7.pdf]

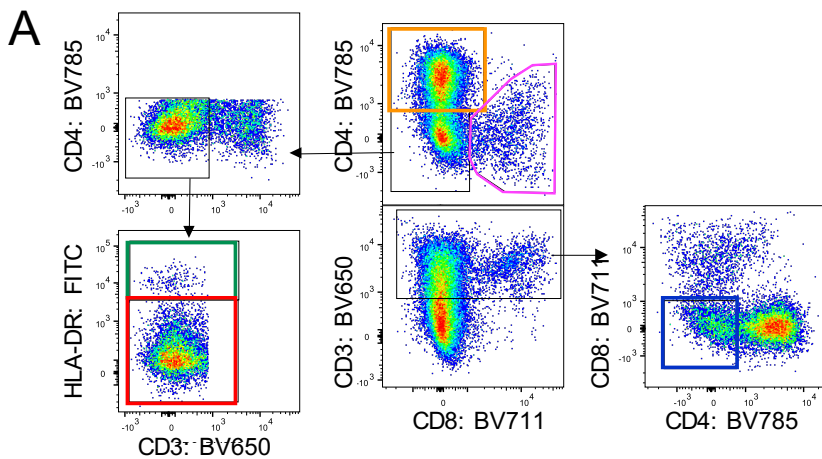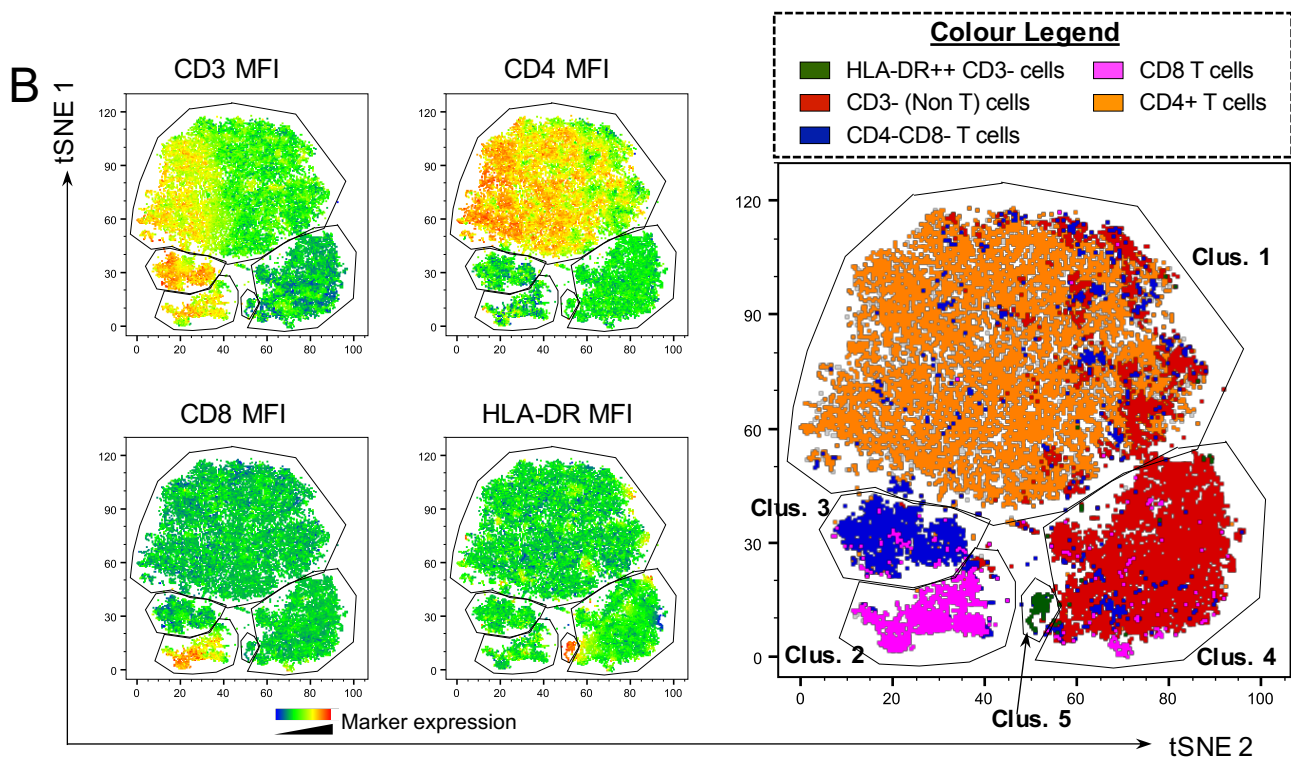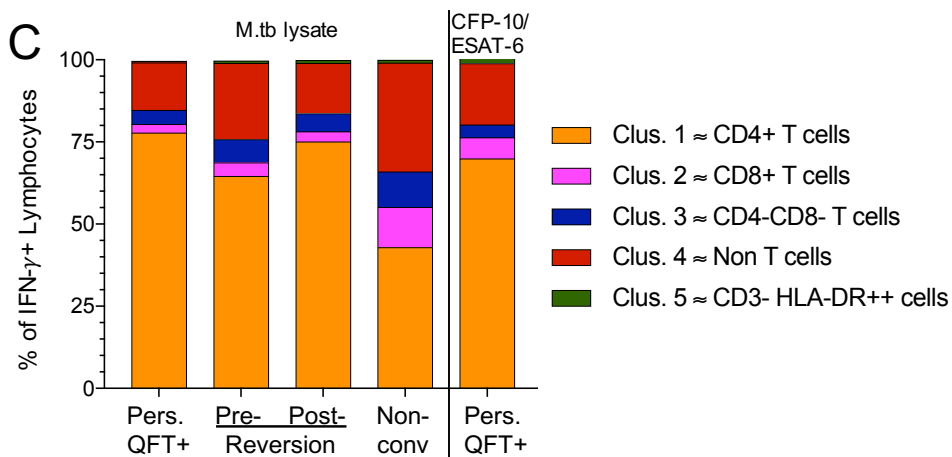

Supplement: Supplementary file 1 [file DataSheet_1.zip › Supp Figure 8.pdf]
