# Supplementary material for: Mycobacterium tuberculosis-Specific T Cell Functional, Memory, and Activation Profiles in QuantiFERON-Reverters Are Consistent With Controlled Infection
Source: Front Immunol. 2021 Aug 30;12:712480. doi: 10.3389/fimmu.2021.712480 (PMC8435731; doi:10.3389/fimmu.2021.712480)
Supplement: Supplementary file 2 [file DataSheet_2.zip › Data Sheet 2/SupplTables/Supp Tab1.docx]

**Supplementary Table 1: Cohort Demographics**

|  | **Persistent QFT+** | **QFT reverters** | **Non-converters** |
| --- | --- | --- | --- |
| N | 30 | 30 | 30 |
| Female sex | 21 | 21 | 21 |
| Median age in years at enrolment  (Range) | 15  (13-18) | 15  (13-18) | 15  (13-18) |
| Ethnicity: Diverse ethnic heritage | 26 | 25 | 25 |
| Ethnicity: Black | 4 | 5 | 5 |
| School D | 15 | 14 | 14 |
| School E | 11 | 11 | 11 |
| School F | 4 | 5 | 5 |
| Known TB exposure (ever) | 7 | 6 | 4 |
| Known TB exposure (<1 year at enrolment) | 4 | 3 | 0 |
| Median body mass index  (Range) | 20.72  (14.70-28.67) | 22.14  (15.70-36.59) | 20.24  (16.24-56.80) |
