# Supplementary material for: Mycobacterium tuberculosis-Specific T Cell Functional, Memory, and Activation Profiles in QuantiFERON-Reverters Are Consistent With Controlled Infection
Source: Front Immunol. 2021 Aug 30;12:712480. doi: 10.3389/fimmu.2021.712480 (PMC8435731; doi:10.3389/fimmu.2021.712480)
Supplement: Supplementary file 2 [file DataSheet_2.zip › Data Sheet 2/SupplTables/Supp Tab2.docx]

**Supplementary Table 2:Adaptive PBMC-ICS flow cytometry panel**

| **Marker** | **Role** | **Fluorochrome** | **Clone** | **Manufacturer** | **Cat. Number** |
| --- | --- | --- | --- | --- | --- |
| CD3 | Lineage | BV650 | UCHT1 | BD Biosciences | 563852 |
| CD4 |  | BV785 | OKT4 | BioLegend | 317442 |
| CD8 |  | BV711 | RPA-T8 | BioLegend | 301044 |
| CCR7 | T cell differentiation | PE | 150503 | BD Biosciences | 560765 |
| CD27 |  | BV510 | L128 | BD Biosciences | 563092 |
| CD45RA |  | BV570 | HI100 | eBioscience | 304132 |
| KLRG-1 |  | PercP-eFlour710 | 13F12F2 | eBioscience | 46948842 |
| CXCR3 | T_SCM_; Homing | PE-Cy5 | 1C6/CXCR3 | BD Biosciences | 551128 |
| HLA-DR | Activation | FITC | L243 | BD Biosciences | 307604 |
| CD107 | Function | PE-CF594 | H4A3 | BD Biosciences | 562628 |
| CD154 (CD40L) |  | BV421 | TRAP-1 | BD Biosciences | 563886 |
| IFN-γ |  | Alexa Fluor 700 | B27 | BD Biosciences | 557995 |
| IL-2 |  | APC | MQ1-17H12 | BD Biosciences | 554567 |
| TNF |  | PE-Cy7 | Mab11 | eBioSciences | 25734982 |
| Live/Dead | Viability | Near IR (APC-H7) | N/A | Life Technologies | L34976 |
