# Supplementary material for: Mycobacterium tuberculosis-Specific T Cell Functional, Memory, and Activation Profiles in QuantiFERON-Reverters Are Consistent With Controlled Infection
Source: Front Immunol. 2021 Aug 30;12:712480. doi: 10.3389/fimmu.2021.712480 (PMC8435731; doi:10.3389/fimmu.2021.712480)
Supplement: Supplementary file 2 [file DataSheet_2.zip › Data Sheet 2/SupplTables/Supp Tab3.docx]

**Supplementary Table 3:Innate PBMC-ICS flow cytometry panel**

| **Marker** | **Role** | **Fluorochrome** | **Clone** | **Manufacturer** | **Cat. Number** |
| --- | --- | --- | --- | --- | --- |
| CD3 | Lineage | BV786 | UCHT1 | BD Bioscience | 563852 |
| CD14 |  | PerCpeF710 | 61D3 | eBioscience | 46014942 |
| CD16 |  | AF488 | 3G8 | Biolegend | 302022 |
| CD19 |  | BV711 | SJ25C1 | BD Bioscience | 563036 |
| CD26 |  | BV605 | M-A261 | BD Bioscience | 744450 |
| CD56 |  | BV50 | HCD56 | Biolegend | 318330 |
| CD161 |  | PECy5 | DX12 | BD Bioscience | 551138 |
| γδ TCR |  | BV421 | 11f2 | BD Bioscience | 744870 |
| GrB | Function | BV510 | GB11 | BD Bioscience | 563388 |
| IL-10 |  | PE-CF594 | JES3-19F1 | BD Bioscience | 562400 |
| IL-6 |  | PE | MQ2-13A5 | BD Bioscience | 554545 |
| IL-12 |  | APC | C11.5 | BD Bioscience | 55457 |
| IFN-γ |  | AF700 | B27 | BD Bioscience | 557995 |
| TNF |  | PECy7 | MAb11 | eBioscience | 25734982 |
| Live/Dead | Viability | Near-IR  (APC-H7) | N/A | Life Technologies | L34976 |
