# Supplementary material for: Mycobacterium tuberculosis-Specific T Cell Functional, Memory, and Activation Profiles in QuantiFERON-Reverters Are Consistent With Controlled Infection
Source: Front Immunol. 2021 Aug 30;12:712480. doi: 10.3389/fimmu.2021.712480 (PMC8435731; doi:10.3389/fimmu.2021.712480)
Supplement: Supplementary file 2 [file DataSheet_2.zip › Data Sheet 2/SupplTables/Supp Tab4.docx]

**Supplementary Table 4: QFT and TST concordance**

|  | **Persistent QFT+** | **QFT reverters** | **Non-converters** |
| --- | --- | --- | --- |
| N | 30 | 30 | 30 |
| Baseline TST [Positive; (Unknown)] | 26; (2) | 17; (4) | 2; (2) |
| 1 year TST [Positive; (Unknown)] | 26; (3) | 20; (3) | 5 |
| TST vs QFT Concordance # | 95% | 45% | 88% |
| TST vs QFT Concordance: Kappa | 0 | -0.086 | 0 |

Note: #: Concordance calculated using QFT and TST test results obtained at the same study visit.
