# Supplementary material for: Mycobacterium tuberculosis-Specific T Cell Functional, Memory, and Activation Profiles in QuantiFERON-Reverters Are Consistent With Controlled Infection
Source: Front Immunol. 2021 Aug 30;12:712480. doi: 10.3389/fimmu.2021.712480 (PMC8435731; doi:10.3389/fimmu.2021.712480)
Supplement: Supplementary file 2 [file DataSheet_2.zip › Data Sheet 2/SupplTables/Supp Tab5.docx]

**Supplementary Table 5: M.tb-specific IFN-γ+ lymphocyte responder rate**

| Group | CFP-10/ESAT-6 | | | M.tb Lysate | | | EspC/EspF/Rv2348 | | |
| --- | --- | --- | --- | --- | --- | --- | --- | --- | --- |
|  | Total  (n) | Responders (n) | % of Responders | Total  (n) | Responders (n) | % of Responders | Total  (n) | Responders (n) | % of Responders |
| Persistent QFT+ | 29 | **21*** | **72.41*** | 30 | **30*** | **100*** | 30 | **13*** | **43.33*** |
| Pre-Rev | 30 | 5 | 16.67 | 28 | **20*** | **71.43*** | 26 | 4 | 15.38 |
| Post-Rev | 28 | 3 | 10.71 | 27 | **20*** | **74.07*** | 23 | 4 | 17.39 |
| Non-Conv | 28 | 6 | 21.43 | 28 | **17*** | **60.71*** | 27 | 3 | 11.11 |

*Values highlighted in **bold red** are groups that had at least 10 responders and a responder rate of ≥33.33%
