# Supplementary material for: Mycobacterium tuberculosis-Specific T Cell Functional, Memory, and Activation Profiles in QuantiFERON-Reverters Are Consistent With Controlled Infection
Source: Front Immunol. 2021 Aug 30;12:712480. doi: 10.3389/fimmu.2021.712480 (PMC8435731; doi:10.3389/fimmu.2021.712480)
Supplement: Supplementary file 2 [file DataSheet_2.zip › Data Sheet 2/SupplTables/Supp Tab6.docx]

**Supplementary Table 6: M.tb-specific Th1 cytokine+ CD4 T cell responder rate**

| Group | CFP-10/ESAT-6 | | | M.tb Lysate | | | EspC/EspF/Rv2348 | | |
| --- | --- | --- | --- | --- | --- | --- | --- | --- | --- |
|  | Total (n) | Responders (n) | % of Responders | Total (n) | Responders (n) | % of Responders | Total (n) | Responders (n) | % of Responders |
| Persistent QFT+ | 29 | **28*** | **96.55*** | 30 | **30*** | **100*** | 30 | **16*** | **53.33*** |
| Pre-Rev | 30 | **11*** | **36.67*** | 27 | **22*** | **81.48*** | 26 | 8 | 30.77 |
| Post-Rev | 29 | **12*** | **41.38*** | 27 | **25*** | **92.59*** | 23 | 6 | 26.09 |
| Non-Conv | 29 | 4 | 13.79 | 29 | **24*** | **82.76*** | 28 | 5 | 17.86 |

*Values highlighted in **bold red** are groups that had at least 10 responders and a responder rate of ≥33.33%.
