# Supplementary material for: Mycobacterium tuberculosis-Specific T Cell Functional, Memory, and Activation Profiles in QuantiFERON-Reverters Are Consistent With Controlled Infection
Source: Front Immunol. 2021 Aug 30;12:712480. doi: 10.3389/fimmu.2021.712480 (PMC8435731; doi:10.3389/fimmu.2021.712480)
Supplement: Supplementary file 2 [file DataSheet_2.zip › Data Sheet 2/SupplTables/Supp Tab7.docx]

**Supplementary Table 7: Memory-cytokine expression profiles of differentially expressed CITRUS clusters in each comparison.**

| **Comparison** | **Antigen-specificity** | **Cluster** | **CD45RA** | **CCR7** | **CD27** | **KLRG-1** | **IFN-𝛾** | **TNF** | **IL-2** |
| --- | --- | --- | --- | --- | --- | --- | --- | --- | --- |
| Persistent QFT+ vs  Pre-reverter | CFP-10 /ESAT-6 | 3718 | 0.51 | 48 | 85.2 | 0.51 | 3.57 | 100 | 0.51 |
|  |  | 3725 | 0 | 12.4 | 42.7 | 21 | 0 | 99.3 | 4.87 |
|  |  | 3714 | 0.43 | 13.7 | 62.4 | 11.1 | 1.28 | 100 | 95.3 |
|  |  | 3711 | 2.8 | 7.48 | 86.9 | 23.4 | 100 | 94.9 | 67.3 |
|  |  | 3707 | 0.43 | 6.01 | 70.4 | 0 | 100 | 100 | 91.8 |
|  |  | 3717 | 0.33 | 1 | 60.5 | 58.5 | 100 | 100 | 99 |
|  |  | 3719 | 1.05 | 5.26 | 65.8 | 0 | 100 | 90 | 17.4 |
|  | M.tb lysate | 9813 | 0.37 | 41.4 | 66 | 10.1 | 0.84 | 100 | 1.96 |
|  |  | 9820 | 11.2 | 36.7 | 78.2 | 7.2 | 4.03 | 79.1 | 96.8 |
|  |  | 9823 | 4.1 | 5.98 | 40 | 41 | 98.3 | 70.8 | 10.8 |
| Pre vs Post- reverter | CFP-10 /ESAT-6 | 932 | 12.5 | 55 | 86.2 | 9.17 | 2.92 | 99.6 | 10.8 |
| Post-reverters vs Non-converter | M.tb lysate | 2662 | 84.8 | 98.9 | 98.9 | 0.56 | 0 | 100 | 3.37 |
|  |  | 2691 | 0.44 | 7.52 | 54.3 | 32.3 | 99.3 | 95.1 | 82.5 |

Numbers denote expression of each memory and functional marker as percentage (%) of total cells in the CITRUS cluster. Markers expressed in less than 35% and more than 65% of total cells denoted in **blue** and **red**, respectively, were used to define the manual gating booleans applied on all participants.
