# Supplementary material for: Mycobacterium tuberculosis-Specific T Cell Functional, Memory, and Activation Profiles in QuantiFERON-Reverters Are Consistent With Controlled Infection
Source: Front Immunol. 2021 Aug 30;12:712480. doi: 10.3389/fimmu.2021.712480 (PMC8435731; doi:10.3389/fimmu.2021.712480)
Supplement: Supplementary file 2 [file DataSheet_2.zip › Data Sheet 2/SupplTables/Supp Tab8.docx]

**Supplementary Table 8: Number of differentially expressed clusters identified between 2 groups using CITRUS.**

| Antigen Specificity | TBI Comparison | Total Clusters | Differentially expressed (DE) clusters | Unique DE populations | Cell populations confirmed by Manual Gating in FlowJo* |
| --- | --- | --- | --- | --- | --- |
| M.tb  lysate | Persistent QFT+ vs Pre-reverter | 31 | 6 | 3 | 3 |
|  | Pre- vs Post-reverter | 30 | 0 | 0 | 0 |
|  | Post-reverter vs Non-converter | 32 | 5 | 2 | 1 |
| CFP-10/  ESAT-6 | Persistent QFT+ vs Pre-reverter | 31 | 21 | 7 | 6 |
|  | Pre- vs Post-reverter | 31 | 1 | 1 | 1 |

Only cell populations that comprised ≥ 2% of antigen-specific responses in all responders were confirmed using manual gating and are represented in Figure 3.
