# Supplementary material for: Mycobacterium tuberculosis-Specific T Cell Functional, Memory, and Activation Profiles in QuantiFERON-Reverters Are Consistent With Controlled Infection
Source: Front Immunol. 2021 Aug 30;12:712480. doi: 10.3389/fimmu.2021.712480 (PMC8435731; doi:10.3389/fimmu.2021.712480)
Supplement: Supplementary file 3 [file DataSheet_3.pdf]

frontiers

Supplementary Online Method:

Mycobacterium tuberculosis-specific T cell functional, memory and activation profiles in QuantiFERON-reverters are consistent with controlled infection.

Innate PBMC stimulation and staining protocol

Stimulation: Cells were thawed, rested for 2 hours and stimulated in R10 media [RPMI 1640 Media (Gibco), 10% Fetal Bovine Serum (FBS, Gibco), 1% L-glutamine (Gibco) and 1% penicillin-streptomycin (Gibco)] with either no antigen (unstimulated, negative control), M.tb lysate (H37Rv 10μg/mL, BEI Resources) or *E.coli* (positive control). Cells were incubated for 2 hours at 37°C, after which brefeldin A (5μg/mL, Sigma Aldrich) and monensin (2.5μg/mL, Sigma Aldrich) were added and incubated for a further 4 hours.

Staining (Supplementary Table 2): Cells were treated with 1mL of 2mM Ethylenediaminetetraacetic acid [EDTA, Sigma Aldrich, in phosphate buffer saline (PBS), Lonza] to detach adherent cells, followed by centrifugation. Supernatant was discarded. Cells were stained in 96-well plates with an antibody cocktail of viability and surface markers in BD Brilliant Stain Buffer (BD Biosciences) for 30 minutes at room temperature. This was followed by a wash in 2% FBS 2mM EDTA (in PBS), fixation and permeabilisation of cells using CytoFix/CytoPerm (BD Biosciences) prior to intra-cellular staining with antibodies for functional markers for 30 minutes at room temperature. Cells were then washed in Perm/Wash (BD Biosciences) and fixed in 1% paraformaldehyde (Kimix) in PBS prior to acquisition on a LSRII flow cytometer (BD Biosciences; Supplementary Figure 6).

Data Analysis Pipeline for Adaptive T cell panel

tSNE analysis of IFN-γ+ lymphocytes

We performed tSNE analysis on IFN-γ+ lymphocytes from responders only to determine the main cellular source of IFN-γ (Supplementary Figure 8). To get one representative FCS file for each sub-group (i.e. non-converter, pre-reverter, post-reverter, persistent QFT+), we first set a maximum threshold count of 500 events per participant and concatenated FCS files from each participant based on QFT status and group. We then sampled all equal events (n ≅ 7000 counts) according to QFT status-

group combination, concatenated all events into one file and performed tSNE analysis on a single FCS file that included all responders from the entire cohort (Supplementary Figure 4 and 5A).

CITRUS analysis of Th1 cytokine+ CD4 T cells

To determine which T cell features were detectable at significantly different abundance levels between groups, we performed CITRUS analysis as described in detail [(2); Supplementary Figure 5B]. Briefly, CITRUS identified clusters of memory and functional CD4 T cell features that were expressed with significantly different (FDR < 0.01) abundance between groups (Supplementary Table 7). Composition of T cell features was then confirmed by manual gating in FlowJo in all responders. Confirmatory statistical tests were performed on manually gated populations that comprised ≥ 2% of antigen-specific responses in all responders. As a result, the final number of cell clusters that were confirmed by manual gating for some the of the comparisons were less than the number of distinct populations identified in CITRUS (Supplementary Table 8).

Data Analysis Pipeline for innate cell panel

We identified IFN-γ+ expressing innate and DURT cell subsets using tSNE. Detectable IFN-γ+ lymphocytes from all individuals were concatenated for each subgroup (persistent QFT+, pre-reverters, post-reverters and non-converters) and each stimulation (unstimulated, M.tb and *E. coli*). FSC files were down sampled to obtain equal events for each subgroup. Down sampled FSC files were concatenated to a single FSC including all subgroups with equal event numbers file for tSNE analysis.

For tSNE including all cytokine+ innate and DURT cells, we included cells expressing IFN-γ, IL-6, TNF, IL-10, IL-12 or granzyme B (GrB) in combination with any other (single GrB expressing lymphocytes were excluded from tSNE analysis) after M.tb stimulation. Available data from all visits were included for all study groups.

Data pre-processing steps applied to the integrated dataset

Data integration: Integration was performed by aligning the innate and adaptive datasets according to participant ID, cohort (QFT reverter, persistent QFT positive or non-converter) and stimulation.

Data standardization: We employed variance stabilizing (vast) scaling to standardize the two datasets to a common scale. Vast scaling is achieved by multiplying the *Z*-score, which was calculated for each variable separately, by the sample mean of that variable as a fraction of the sample standard deviation.

Missing value imputation: Missing values in the integrated dataset were meaningfully replaced using a multiple factor analysis (MFA)-based imputation method (3). The imputation method was performed on the vast scaled integrated dataset within the missMDA R package (R package version 1.16.; https://cran.r-project.org/web/packages/missMDA/), and we defined the groups as the variables that came from the different datasets.

Supplementary Equation 1: Functional Differentiation Score.

$$FDS = \frac{\boldsymbol{IFN - \gamma} + TNF \pm IL - 2 \pm}{\boldsymbol{IFN - \gamma} - TNF \pm IL - 2 \pm}$$

<u>Supplementary Figure Legends:</u>

Supplementary Figure 1: QFT results. Graphs illustrate QFT responses measured biannually during follow-up in adolescent persistent QFT+, reverters and non-converters. Positive and negative QFT responses are depicted by red and blue symbols, respectively. To plot all data points on a log scale we set values equal to 0 to 0.01. Dotted line and shaded area represent the QFT assay threshold (0.35 IU/mL) and uncertainty zone (0.2-0.7 IU/mL), respectively.

Supplementary Figure 2: Adaptive T cell panel gating strategy. To identify M.tb-responsive cells in the PBMC-ICS assay, we first gated on total cells acquired consistently (time gate), followed by gating on singlets, live cells and exclusion of antibody aggregates. We then gated on total lymphocytes and excluded additional antibody aggregates. Due to the major down-regulation of CD3 expression on cytokine-expressing cells, we first gated on CD4+ (after excluding CD8+ cells) and CD8+ (after excluding CD4+ cells) T cells and then gated on CD3+ T cells. Functional markers (IFN-γ+, TNF, IL-2, CD154 and CD107) were then gated independently on CD4+CD3+ (blue box) and CD8+CD3+ (green box) T cells, and total lymphocytes (purple box).

Supplementary Figure 3: Calculation of median responses based on QFT status. Kruskal-Wallis test was performed on functional responses from (A) persistent QFT+ individuals and (B) non-converters, and Wilcoxon-signed rank tests were performed on functional responses from (C) reverters, to determine whether serial immune responses were significantly different based on QFT status (positive or negative). To plot all data points, frequencies of IFN-γ+ CD4 T cells below 0.0001% were set to 0.0001%. This analysis was repeated for all variables before calculating the median values for each participant across multiple visits with the same QFT result.

Supplementary Figure 4: Analysis pipeline used to define a single FCS file per participant-QFT status. (1) Cytokine+ total lymphocytes and CD4 T cells were gated in FlowJo and counts exported to determine M.tb-specific responders. (2) Responders to antigenic stimulation were defined as IFN-γ+ lymphocytes and/or total Th1 cytokine+ CD4 T cell responses for each participant at each visit that had a MIMOSA FDR ≤ 0.01 and a fold change of antigen-specific signal over unstimulated of ≥ 3. (3) IFN-γ+ lymphocytes and/or total Th1 cytokine+ FCS files for all participant visits that passed the responder criteria were then exported, and (4) visits were concatenated based on the participant QFT-status.

Supplementary Figure 5: Lymphocyte and CD4 T cells Analysis Pipeline. (A) (i) IFN-γ+ lymphocyte FCS files were down-sampled to a maximum of 500 cell counts per participant-QFT status, (ii) all FCS files from participants belonging to the same QFT-group were concatenated into one FCS file per group. (iii) Equal number of cells (n=7000) from each group were then concatenated into one file, (iv) which was used to perform tSNE analysis. (B) (i) Total Th1 cytokine+ FCS files from all individuals were imported into Cytobank to conduct (ii) CITRUS analysis between groups (# 2 groups were compared for each CITRUS analysis: persistent QFT+ *versus* pre-reverter, pre- *versus* post-reverter and post-reverter *versus* non-converter). (iii) Differentially expressed clusters (FDR < 0.01) identified in CITRUS, were then exported from CITRUS and concatenated in to one file per cluster. (iv) Gates were set in FlowJo to confirm patterns of marker expression that defined each cluster. (v) Final gating strategy of each cluster was then applied to individual responders, and (vi) confirmatory statistical analysis was performed on manually gated populations.

Supplementary Figure 6: Innate panel gating strategy. To identify M.tb-responsive cells in the PBMC-ICS assay, we first gated on total cells acquired consistently (time gate), followed by gating on live cells, singlets and exclusion of antibody aggregates. Representative examples of cytokine

expression are shown for the different cell subsets in unstimulated samples as well as upon M.tb lysate and *E. coli* stimulation.

Supplementary Figure 7: TST results. TST induration measured during follow-up in persistent QFT+ individuals, reverters and non-converters. Positive and negative QFT responses are depicted by red and blue symbols, respectively. TST cut-off of 5mm, as defined by (1), is represented by the dotted line.

Supplementary Figure 8: CD4 T cells are the main source of IFN-γ in QFT+ individuals. (A) Flow cytometry plots depict manual gating of CD4+ T cells (orange), CD8+ T cells (pink), CD4-CD8- T cells (blue), HLA-DR++ CD3- cells (green) and CD3-CD4-CD8- (non T) cells (red) on total IFN-γ+ lymphocytes. (B) Median fluorescence intensity (MFI) of CD3, CD4, CD8 and HLA-DR is shown on the tSNE maps on the left. On the right, manually gated population in (A) are superimposed to the 5 cell clusters identified in tSNE. (C) Relative proportions of the 5 cell clusters contributing to the total IFN-γ+ lymphocytes in response to M.tb lysate and CFP-10/ESAT-6 in the study groups. Robust total lymphocyte responses to CFP-10/ESAT-6 were detected in persistent QFT+ only (Supplementary Table 5).

Supplementary Figure 9: QFT reverters maintain expression of functional markers other than IFN-γ. (A) Frequencies of background subtracted CFP-10/ESAT-6-specific total TNF+, IL-2+, CD154+ and CD107+ CD4 T cells, and (B) median frequencies of CFP-10/ESAT-6-cytokine co-expressing CD4 T cells detected in persistent QFT+ (n=29, white), pre-reversion (n=30, red), post-reversion (n=28, blue) and non-converters (n=28, grey). P-values were calculated as in Figure 1, with p-values considered significant after correction for multiple comparisons highlighted in **bold** red.

Supplementary Figure 10: Reverters have lower CFP-10/ESAT-6-specific functional differentiation than persistent QFT+ individuals. Median proportions of M.tb-specific Th1 cells expressing (A) different cytokine combinations and (B) different combinations of memory markers in response to stimulation with M.tb lysate, CFP-10/ESAT-6- and EspC/EspF/Rv2348 in persistent QFT+ (P10-ES6: n=28; M.tbL: n= 30; Esp: n=16), pre-reversion (P10-ES6: n=11; M.tbL: n= 22), post-reversion (P10-ES6: n=12; M.tbL: n= 25) and non-converters (M.tbL: n= 24). P-values were calculated as in Figure 1, with p-values considered significant after correction for multiple comparisons highlighted in **bold** red.

Supplementary Figure 11: IFN-γ expressing lymphocytes. (A) tSNE visualization of IFN-γ+ lymphocyte subsets after *M.tb* lysate or *E. coli* stimulation and unstimulated samples from all participants (innate protocol). Manually gated cell subsets (Supplementary Figure 6) are overlayed on the tSNE plots. (B) Proportions of different cell subsets contributing to the total IFN-γ production in each study group were calculated as in Supplementary Figure 8C.

Supplementary Figure 12: Cytokine expression in innate and DURT cells. tSNE visualization of the cellular source and cytokine expressed in each cluster in response to M.tb lysate stimulation. Manually gated cell subsets or functions (Supplementary Figure 6) are overlayed on the tSNE plot. All participants and all visits are shown.

Supplementary Figure 13: PLS-DA model built to the LASSO feature selected variables. (A) Univariate plot of the loading scores of each group on latent component two, where the pre- and post-reverter groups were compared using Wilcoxon's signed rank paired test and the other groups were compared using Mann Whitney's U test. The resulting p-values are superimposed onto the plot. (B) Feature importance on latent component 2. (C) Loading scores for the PLS-DA model applied to reverters who were classified as responders or non-responders to CFP-10/ESAT-6 stimulation (Supplementary Table 6). The loading scores in (C) were calculated separately from (A) and (B).

References

1. Mpande CAM, Rozot V, Mosito B, Musvosvi M, Dintwe OB, Bilek N, et al. Immune profiling of Mycobacterium tuberculosis-specific T cells in recent and remote infection. *Ebiomedicine* (2021) **64**:103233. doi:10.1016/j.ebiom.2021.103233

2. Husson F, Josse J. Handling missing values in multiple factor analysis. *Quality and Preferences* (2013) **30**:77–85.

3. Mahomed H, Hawkridge T, Verver S, Geiter L, Hatherill M, Abrahams D-A, et al. Predictive factors for latent tuberculosis infection among adolescents in a high-burden area in South Africa. *Int J Tuberc Lung Dis Official J Int Union Against Tuberc Lung Dis* (2011) **15**:331–6.
